# Targeting of DDR1 with antibody‐drug conjugates has antitumor effects in a mouse model of colon carcinoma

**DOI:** 10.1002/1878-0261.12520

**Published:** 2019-07-22

**Authors:** Yiran Tao, Ruixue Wang, Qinhuai Lai, Mengdan Wu, Yuxi Wang, Xiaohua Jiang, Lishi Zeng, Shijie Zhou, Zhongping Li, Tinghan Yang, Yuqin Yao, Yangping Wu, Lin Yu, Yuyin Fu, Weirong Lai, Yujia Peng, Ying Lu, Zhixiong Zhang, Cuiyu Guo, Guangbing Zhang, Lantu Gou, Jinliang Yang

**Affiliations:** ^1^ State Key Laboratory of Biotherapy and Cancer Center/Collaborative Innovation Center for Biotherapy West China Hospital Sichuan University Chengdu China; ^2^ Department of Respiratory and Critical Care Medicine West China Hospital Sichuan University Chengdu China; ^3^ Department of Gastrointestinal Surgery West China Hospital Sichuan University Chengdu China; ^4^ West China School of Public Health and Healthy Food Evaluation Research Center/No. 4 West China Teaching Hospital Sichuan University Chengdu China; ^5^ Department of Clinical Research Management West China Hospital Sichuan University Chengdu China; ^6^ Department of Clinical Laboratory Mianyang Central Hospital Mianyang China; ^7^ Guangdong Zhongsheng Pharmaceutical Co., Ltd. Dongguan China

**Keywords:** antibody‐drug conjugate, colon cancer, receptor tyrosine kinase, resistance, xenograft tumor model

## Abstract

DDR1 has been identified as a cancer‐associated receptor tyrosine kinase that is highly expressed in several malignancies relative to normal tissues. Clinically approved multi‐kinase inhibitors, such as nilotinib, inhibit DDR1‐mediated tumor growth in xenograft models, suggesting DDR1 might be a potential target for cancer treatments. Here, we employed an antibody‐based strategy with a novel anti‐DDR1 antibody‐drug conjugate (ADC) for colon carcinoma treatment. We developed T_4_H_11_‐DM4, an ADC targeting DDR1 which carries the tubulin inhibitor payload DM4. Immunohistochemical analysis of a tissue microarray containing 100 colon cancer specimens revealed that DDR1 was highly expressed in 81% of tumor tissues. Meanwhile, high expression of DDR1 was associated with poor survival in patients. *In vitro*, T_4_H_11_‐DM4 exhibited potent anti‐proliferative activity with half maximal inhibitory concentration (IC_50_) values in the nanomolar range in a panel of colon cancer cell lines. *In vivo*, the antitumor efficacy of T_4_H_11_‐DM4 was evaluated in three colon cancer cell lines expressing different levels of DDR1. T_4_H_11_‐DM4 achieved complete tumor regression at doses of 5 and 10 mg·kg^−1^ in HT‐29 and HCT116 tumor models. Moreover, a correlation between *in vivo* efficacy of T_4_H_11_‐DM4 and the levels of DDR1 expression on the cell surface was observed. Tumor cell proliferation was caused by the induction of mitotic arrest, indicating that the antitumor effect *in vivo* was mediated by DM4. In addition, T_4_H_11_‐DM4 was efficacious in oxaliplatin‐resistant colon cancer models. In exploratory safety studies, T_4_H_11_‐DM4 exhibited no overt toxicities when multi‐doses were administered at 10 mg·kg^−1^ into BALB/c nude mice or when a single dose up to 50 mg·kg^−1^ was administered into BALB/c mice. Overall, our findings highlight the potential of DDR1‐targeted ADC and may facilitate the development of a new effective therapeutic strategy for colon cancer.

**Abbreviations**
ADC: antibody‐drug conjugates
CRC: colorectal carcinoma
DAR: drug‐antibody ratios
DDR: discoidin domain receptor
DM4: N2′‐deacetyl‐N2′‐(4‐mer‐capto‐4‐methyl‐1‐oxopentyl)‐maytansine
ECD: extracellular domain
EGFR: epidermal growth factor receptor
FCM: flow cytometry
IHC: immunohistochemistry
MFI: mean fluorescence intensity
NIR: near‐infrared
OS: overall survival
RTK: receptor tyrosine kinase
SPR: surface plasmon resonance
VEGFR: VEGF receptors
VEGF: vascular endothelial growth factor

## 1 Introduction

Colorectal cancer (CRC) is the third most common cancer globally, with 1.85 million new cases and an estimated 8.8 hundred thousand deaths from the disease in 2018 around the world (Source: Globocan 2018). Owing to advances in the knowledge of the molecular basis of CRC, targeted therapies in combination with radiotherapy and chemotherapy have prolonged progression‐free survival and overall survival (OS) of patients with advanced CRC (Augestad *et al*., 2017). At present, the best‐known therapeutics used in clinic for CRC treatment include bevacizumab, cetuximab and panitumumab, which target vascular endothelial growth factor (VEGF), VEGF receptors (VEGFR) and epidermal growth factor receptor (EGFR) (Falchook and Kurzrock, 2015; Saucier and Rivard, 2010). However, acquired resistance and relapse have often occurred and led to death in the majority of patients after multiagent treatments (Hammond *et al*., 2015). Therefore, there is a strong need for new potential treatment strategies for colon cancer (Van der Jeught *et al*., 2018).

The discoidin domain receptor (DDR) family is a unique set of receptor tyrosine kinases (RTKs) and consists two distinct members, DDR1 and DDR2, which are involved in cell proliferation, adhesion and migration (Gao *et al*., 2016; Rammal *et al*., 2016). DDR1 is found preferentially expressed in highly invasive epithelial tumor cells, whereas DDR2 is expressed in tumor stroma (Borza and Pozzi, 2014; Henriet *et al*., 2018). DDR1 is a single transmembrane receptor and has five isoforms due to the alternatively encoding spliced transcript variants, but their extracellular regions are highly conserved. Upon activation by binding to collagen, DDR1 exhibits sustained receptor phosphorylation and induces several downstream signaling pathways linked to tumor progression in several human cancers.

Recently, DDR1 aberrant expression has been described in different cancer cell lines and cancer patients, such as lung (Ambrogio *et al*., 2016), breast (Friese‐Hamim and Vogel, 2005), esophagus (Nemoto *et al*., 1997), ovary (Heinzelmann‐Schwarz *et al*., 2004; Quan *et al*., 2011) and colon cancers (Weiner *et al*., 2000). These observations suggested that this collagen‐activated RTK is involved in the development and progression of tumors. Overexpression of DDR1 in non‐small lung cancer cells and hepatocellular carcinoma significantly promotes tumor cell motility (Ezzoukhry *et al*., 2016). Genetic inhibition of DDR1 in human colon (Kim *et al*., 2017), glioma (Ram *et al*., 2006; Yamanaka *et al*., 2006) and pancreatic adenocarcinoma carcinoma cells (Aguilera *et al*., 2017) shows impaired growth of tumor xenograft in mice.

Hence DDR1 is considered a promising target for cancer therapy. Several FDA‐approved multi‐target small molecule RTK inhibitors such as imatinib, nilotinib and dasatinib can also block kinase activity of DDR1 with IC_50_ values in the low nanomolar range (Day *et al*., 2008; Rix *et al*., 2007). It has been shown that nilotinib strongly reduced DDR1‐mediated CRC cell invasion and metastasis in mouse models (Jeitany *et al*., 2018). In recent years, a panel of selective DDR1 kinase inhibitors has been developed, such as DDR1‐IN‐1 and 7rh benzamide (Gao *et al*., 2013). *In vivo* experiments showed that 7rh benzamide could slow tumor growth and induce a 50% suppression of tumor size in subcutaneous xenografts of gastric carcinoma (Hur *et al*., 2017). Besides, monoclonal antibody 48B3 specific to DDR1 could decrease glioma cell invasion and adhesion (Ram *et al*., 2006). Collectively, these results indicate that DDR1 inhibition may provide a therapeutic approach for treating tumors. However, the anti‐tumor efficacy of these DDR1 inhibitors which depend on DDR1 signaling for cancer cells survival is limited to suppress tumor growth and not sufficient to induce complete tumor regression *in vivo*.

Antibody‐drug conjugate (ADC) is a novel strategy for tumor therapy which combines the specificity of monoclonal antibodies to target selectively tumor cells with the potent killing activity of payloads. There are four ADC approved by FDA. Currently, more than 70 ADC are under clinical evaluation. Nearly 175 investigational ADC are in development from early discovery to pivotal stage (Chalouni and Doll, 2018). IMMU‐130, a CEACAM5‐targeted ADC which is now under phase 2 study, showed encouraging results in patients with late‐stage metastatic CRC (Dotan *et al*., 2015).Additionally, other ADC target Lgr5 (Junttila *et al*., 2015), Sialyl‐Thomsen‐nouveau antigen (Prendergast *et al*., 2017), RON RTK (Feng *et al*., 2014) and 5T4 oncotrophoblast glycoprotein (Wang *et al*., 2018) have been under development for CRC treatment.

The DDR1 cell‐surface localization and swift endocytosis characteristics make it a targetable antigen for the ADC and compelled us to assess the potential DDR1‐targeted ADC colon carcinoma therapy. In this study, we developed DDR1 antibody‐DM4 conjugates called T_4_H_11_‐DM4. T_4_H_11_‐DM4 demonstrated potent antitumor efficacy both *in vitro* and *in vivo* with an acceptable safety profile, suggesting anti‐DDR1 ADC is a promising strategy for colon carcinoma therapy.

## 2 Materials and methods

### 2.1 Cell culture

Human HT‐29, HCT116, HCT‐15, Caco‐2, DLD‐1, SW48, SW480, SW620 and LoVo colon carcinoma cell lines; mouse myeloma cell line SP2/0 cells were purchased from the ATCC. Oxaliplatin‐resistant cell lines SW480‐OR and HCT116‐OR were obtained from our laboratory (Wang *et al*., 2018). Human embryonic kidney cell line 293F (HEK293F) was purchased from Life Technologies and cultured in FreeStyle™ 293 Expression Medium (Gibco, Thermo Scientific, Waltham, MA, USA) (Zhang *et al*., 2017). Cell lines were authenticated by Feiouer Bio‐Technique Co., Ltd (Chengdu, China) using short tandem repeat DNA fingerprinting technique. Tumor cell lines were cultured at 37 °C, 5% CO_2_ in a humidified incubator in standard cell culture media as indicated by the provider.

### 2.2 Tissue microarray

DDR1 expression in human normal tissues and colon carcinoma tissues was evaluated using tissue microarrays (TMA; Shanghai Outdo Biotech, Shanghai, China) stained with the anti‐DDR1 antibody (Novus Biologicals, Centennial, CO, USA) by immunohistochemical (IHC) analysis. Paraffin‐embedded TMA were deparaffinized in xylene and rehydrated in gradient concentration of ethanol. After pretreatment with 3% hydrogen peroxide for 150 min, TMA were treated with Retrieval Solution for 15 min at 99 °C and incubated with anti‐DDR1 antibody overnight after blocking with goat serum for 1.5 h at room temperature. Following a wash, slides were incubated with DakoReal™EnVision™ horseradish peroxidase‐conjugated anti‐rabbit antibody for 30 min at room temperature and then visualized using diaminobenzidine (Dako, Carpinteria, CA, USA). Staining was analyzed via Imagescope Viewer (Leica Biosystems, Buffalo Grove, IL, USA). The percentage of positive cells and the strength of staining codetermined staining intensity. Intensity scores were defined as: 0, no detectable staining signal in > 50% of tumor cells; 1+, weak staining signal detected in > 50% of tumor cells; 2+, moderate staining signal in > 50% of tumor cells; 3+, strong staining signal in > 50% of tumor cells. The staining intensity was further dichotomized into score 0/1+ for low DDR1expression or score 2+/3+ for high DDR1 expression.

### 2.3 Flow cytometry

Flow cytometry (FCM; FACS Calibur, BD, Franklin Lakes, NJ, USA) was used to determine DDR1 expression in colon cancer cell lines. Cells were resuspended with PBS with 2 mmol·L^−1^ EDTA. All subsequent steps were carried out at 4 °C. Cells (2 × 10^5^/tube) were incubated with 5 μg·mL^−1^ primary antibody for 40 min, followed by Alexa Fluor 488‐labeled goat anti‐mouse IgG (H+L) secondary antibody (Zsbio, Beijing, China). All samples were washed three times before being analyzed by FCM. To process the obtained data, novoexpress software (ACEA Biosciences, Hangzhou, China) was utilized.

### 2.4 Generation of anti‐DDR1 antibodies

Anti‐DDR1 antibodies were generated by the mouse hybridoma method. Recombinant protein with His‐tag corresponding to the extracellular domain (ECD, amino acids 21–417) of Human DDR1 (His‐DDR1) purified from supernatants of HEK293F cells was utilized as the immunogen. BALB/c mice were immunized with 50 μg of the immunogen in combination with adjuvant. Five days after the final boost, mouse spleen cells were harvested and fused with SP2/0 cells. Hybridoma supernatants were screened by ELISA against the immunogen. Positive cell lines were further screened for binding and internalization abilities by FCM using DDR1 expression cells. Antibodies were purified using protein G (GE Healthcare, Uppsala, Sweden) affinity chromatography.

### 2.5 Preparation of T_4_H_11_‐DM4, T_4_H_11_‐Cy5.5 and IgG‐Cy5.5

The anti‐DDR1 monoclonal antibody (mAb), clone T_4_H_11_ and isotype control IgG (mouse IgG; Bioss, Beijing, China) were used to prepare ADC. T_4_H_11_ or control IgG was mixed with a 10‐fold molar excess of SPDB‐DM4 (Accela ChemBio, Shanghai, China) in conjugation buffer at a concentration of 5 mg·mL^−1^. The coupling reactions were performed overnight at 25 °C. The reaction mixtures were separated by chromatography using a desalting column (GE Healthcare) to yield T_4_H_11_‐DM4 and IgG‐DM4 conjugates. The drug‐antibody ratio (DAR) of T_4_H_11_‐DM4 was confirmed by LC‐MS. Amino‐based bioconjugation method was conducted to prepare T_4_H_11_‐Cy5.5 and control IgG‐Cy5.5 with the method described above.

### 2.6 Biacore

Surface plasmon resonance (SPR)‐based measurements were performed by Biacore T200 (GE Healthcare) instrument. His‐DDR1 ECD protein was captured via an NTA sensor chip by Ni^2+^ chelation according to the manufacturer's instructions prior to capture of antibodies. For kinetic analysis, T_4_H_11_ or T_4_H_11_ *‐*DM4 was run across the chip in a 2‐fold dilution series, with another channel set as control. Each sample bound across the antigen surface was dissociated by HBS‐P+ running buffer [10 mmol·L^−1^ HEPES, 150 mmol·L^−1^ NaCl, pH 7.4, 0.05% (v/v) surfactant P20, 0.05 mmol·L^−1^ EDTA] for 300 s at a flow rate of 30 μL·min^−1^. Regeneration of the sensor chips was performed for 60 s using regeneration buffer (350 mmol·L^−1^ EDTA).The association and dissociation rate constants *k*_a_ and *k*_d_ were monitored respectively and the affinity value *K*_D_ was determined. To determine the kinetic binding parameters from which affinities are calculated, biacore t200 evaluation software 3.0 (GE Healthcare, Uppsala, Sweden) was used.

### 2.7 ELISA

ELISA plates were coated with 1 μg·mL^−1^ (100 μL per well) of His‐DDR1 or His‐DDR2 extracellular antigen in PBS and dried overnight before blocking with PBS/2% BSA (Biosharp, Hefei, China). Antibody (0.00375 μg·mL^−1^–10 μg·mL^−1^) was added and incubated at 37 °C for 2 h. Plates were washed three times with 200 μL PBS/Tween 20 (0.05%). HRP‐goat anti‐mouse IgG (Proteintech, Wuhan, Hubei, China) was added and left at 37 °C for 40 min. Following this, a further four washes in PBS/Tween 20 (0.05%) were carried out before adding 100 μL per well of TMB substrate. The reaction was stopped with 2 mol·L^−1^ sulfuric acid and reading at 450 nm using an ELISA plate reader.

### 2.8 FCM to determine cell binding ability

For determination of *in vitro* binding ability, cells (2 × 10^5^/tube) were incubated with dilution concentrations ranging from 0.0187 to 40 μg·mL^−1^ of primary antibodies for 40 min each at 4 °C followed by Alexa Fluor 488‐labeled goat anti‐mouse IgG(H+L) secondary antibody (Zsbio). Mean fluorescence intensity (MFI) of detectable binding of antibodies over multiple test concentrations was measured by FCM. MFI values of samples were subtracted from their respective negative control antibodies and analyzed using the nonlinear regression analysis in prism® software version 5 (Graphpad Software, San Diego, CA, USA).

### 2.9 Confocal microscopy detection

Internalization of anti‐DDR1 mAb was detected by confocal microscopy and FCM, respectively. For confocal microscopy experiment, HT‐29 cells were seeded onto slides at a density of 5 × 10^3^ cells·mL^−1^ for 24 h. Thee media were then replaced with fresh medium containing DDR1 mAb labeled with fluorescein (495/520 nm) according to the manufacturer's protocol (Dojindo, Shanghai, China). After 40 min incubation on ice, cells were washed to remove excess antibodies with PBS and the experimental group was incubated for 3 h at 37 °C to conduct internalization while the control group was kept at 4 °C. Cells were washed after internalization. 4,6‐Diamidino‐2‐phenylindole (Beyotime, Shanghai, China) was added to stain nuclei for 5 min. The slide was placed on a microscope slide and mounted by applying antifading medium (Beyotime). Images were acquired on a Confocal Fluorescence Imaging Microscope (Leica TCS‐SP5, Buffalo Grove, IL, USA) and analyzed using Leica application suite 2.02.

### 2.10 Cellular internalization of T_4_H_11_ and T_4_H_11_‐DM4 by FCM

After T_4_H_11_ or T_4_H_11_ *‐*DM4 binding to HT‐29 cells, the experimental group was incubated for 3 h at 37 °C to conduct internalization while the control group was kept at 4 °C. The degree of internalization of cell surface‐bound antibody was determined by the percentage of decrease in MFI of the experimental group compared with the control group. The following formula was used to calculate the internalization efficiency of each antibody in cells: internalization efficiency (%) = [(MFI of the control group − MFI of the experimental group)/MFI of the control group] × 100% (Wang *et al*., 2018).

### 2.11 *In vitro* cell viability assay

Cell viability in the presence of T_4_H_11_, T_4_H_11_ *‐*DM4 or isotype control IgG‐DM4 was evaluated in triplicate by Cell Counting Kit‐8 assays (CCK‐8; Dojindo). All tumor cell lines were plated at log phase of growth in 96‐well plates at a density of 3–5 × 10^3^ cells with 100 μL culture medium. Drugs were serially diluted and added to plates. After 72 h of drug exposure, CCK‐8 was added into the wells to obtain dose‐response curves. The IC_50_ of drugs on tumor cells was calculated using prism®.

### 2.12 *In vivo* fluorescent imaging

A near‐infrared (NIR) fluorochrome, Cy5.5, was conjugated into T_4_H_11_ or control IgG to monitor dynamic distribution and tumor‐targeting capability *in vivo*. HT‐29 xenograft model was established as described in the following section. When tumors reached 150 mm^3^, mice were intravenously injected with Cy5.5‐T_4_H_11_ or Cy5.5‐IgG at 10 mg·kg^−1^. Then tumor‐bearing mice were anesthetized by inhalation of isoflurane and imaged at various time points post injection. Exposure time was 0.25 s per image. At predetermined time points, fluorescence images were obtained using an IVIS Lumina imaging® system (Perkin Elmer, Waltham, MA, USA) at 675 nm for excitation and 694 nm for emission. Images were analyzed using living image 4.4 software (Perkin Elmer, Waltham, MA, USA). After *in vivo* imaging, mice were euthanized. Tumors and vital organs were excised and washed with saline, followed by *ex vivo* imaging.

### 2.13 *In vivo* treatment

All *in vivo* treatments were conducted according to The Institutional Animal Care and Treatment Committee of State Key Laboratory of Biotherapy in Sichuan University. BALB/c nude mice (Hfkbio, Beijing, China) were given a single subcutaneous injection of approximately 100 μL of 1 × 10^7^ HT‐29, HCT116, HCT‐15 and SW480‐OR cells. When mean tumor volumes reached approximately 100–200 mm^3^, tumor‐bearing mice were randomized into groups of six (day 1). Mice were administered three doses of T_4_H_11_ *‐*DM4 (10, 5 and 2.5 mg·kg^−1^, 100 μL), unconjugated T_4_H_11_ (10 mg·kg^−1^, 100 μL) and vehicle (PBS, 100 μL) once, 3 days intravenously. Tumor size was measured using calipers. Mice were weighed to assess the toxicity of treatment. Tumor volume [(length × width^2^)/2] and mice weight were measured at least twice a week. Mice were sacrificed when tumors reached a mean volume of 2000 mm^3^. Complete tumor regression was defined as no palpable tumor detected.

### 2.14 Immunohistochemistry

Following formalin fixing, paraffin‐embedded xenografted tumor tissue sections were deparaffinized in xylene and rehydrated with decreasing grades of ethanol. The slides were preincubated with 3% hydrogen peroxide methanolic solution for 30 min at room temperature and heat‐induced epitope retrieval was carried out. After blocking with goat serum for 2 h, slides were incubated with rabbit anti‐DDR1 (Novus Biologicals) 1 : 100 diluted at 4 °C overnight. Following a wash, slides were incubated with horseradish peroxidase‐conjugated anti‐rabbit antibody (Zsbio) for 30 min at 37 °C and then visualized using diaminobenzidine tetrahydrochloride.

### 2.15 Statistical analysis

Statistical analysis was performed using prism® software version 5 (GraphPad Software, Inc.). OS data were analyzed by the Kaplan–Meier method. Survival curves were generated based on log‐rank test. Results are shown as mean ± SD. Bars exhibited on vertical scatter plots represent the geometric mean or mean for each group. *P* values < 0.05 were considered to be significant in this study.

## 3 Results

### 3.1 DDR1 is highly expressed on the surface in tissues and cell lines of colon cancer

**Figure 1:**
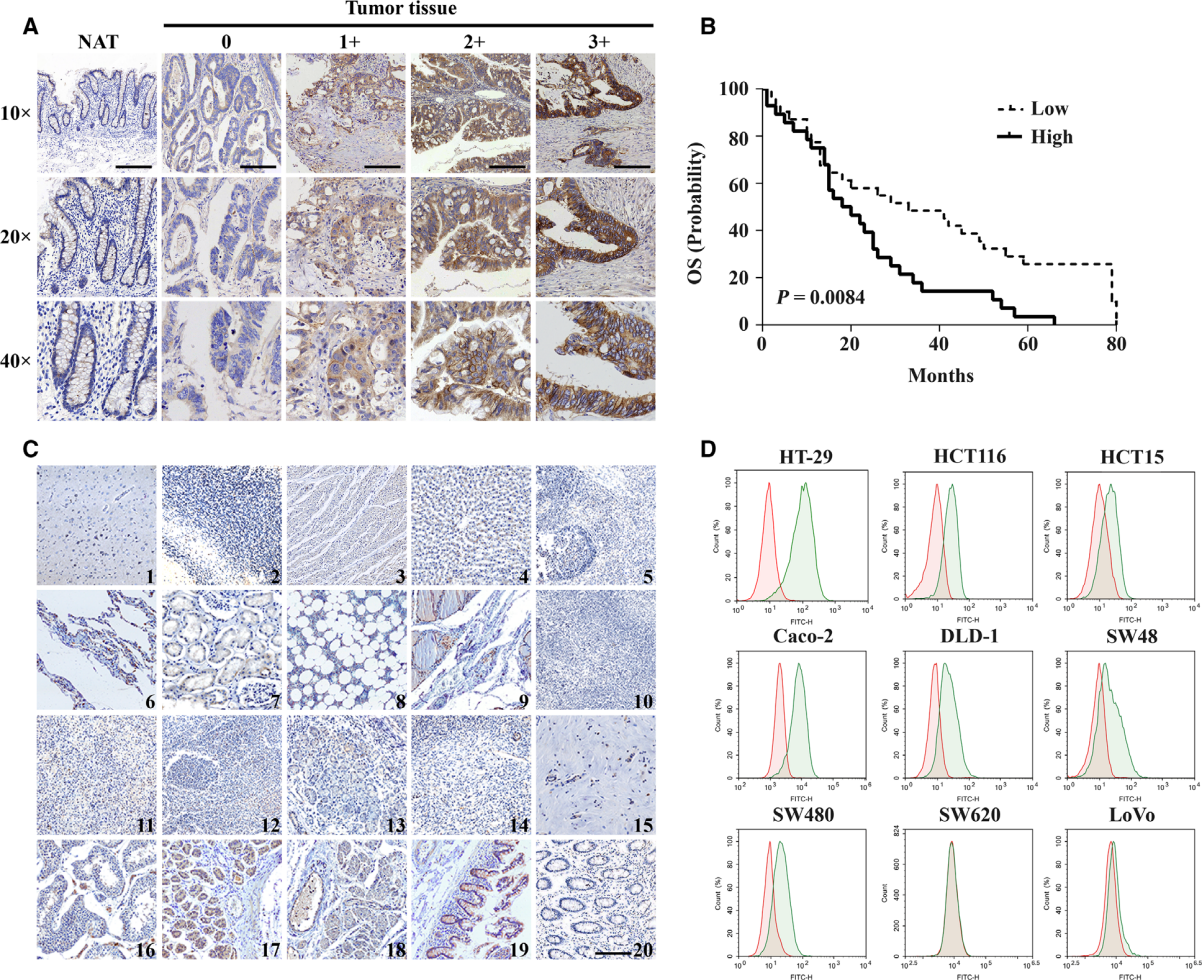
Expression of DDR1 in human tumor and normal tissues. (A) Representative images of IHC staining for DDR1 expression in normal adjacent tissues (NAT) and cancerous tissues of colon cancer patients (*n* = 100), representing increasing intensity of staining. Samples were stained for DDR1 antigen (brown stain in membrane or cytoplasm) and counterstained with hematoxylin (blue stain in nucleus). Representative images: 0, no detectable staining intensity; 1+, weak staining intensity; 2+, moderate staining intensity; 3+, strong staining intensity. Black scale bar: 250 μm. (B) Kaplan–Meier survival curves depicting OS in colon cancer patients with high and low DDR1 expression. (C) Representative images of IHC staining for DDR1 in human normal tissues. 1, cerebrum; 2, cerebellum; 3, heart; 4, liver; 5, spleen; 6, lung; 7, kidney; 8, bone marrow; 9, thymus gland; 10, lymph node; 11, adrenal gland; 12, tonsil; 13, tongue; 14, ovary; 15, breast; 16, testis, 17, stomach; 18, esophagus; 19, small intestine; 20, colon. Magnification, ×10. Black scale bar: 250 μm. (D) FCM analysis of DDR1 cell surface expression in a panel of colon cell lines. Cells detected with an isotype IgG (red) or anti‐DDR1 monoclonal antibody (green), respectively.

To assess the prevalence of DDR1 expression, human colon cancer tissue and normal TMAs were evaluated by IHC staining. The results showed DDR1 staining was positive in 94% (94/100) of cancer samples. Of the 100 tumor tissues, 81 showed moderate (48%) to strong (33%) expression of DDR1, scoring as 2+ or 3+. In contrast, weak or no staining was found in normal adjacent colon cancer tissue (Fig. 1A). DDR1 was expressed on both cell membranes and cytoplasm in colon cancer. Furthermore, OS was significantly shorter in the high DDR1 expression group than the low DDR1 expression group of patients with colon cancer (*P* = 0.0084, Fig. 1B). We next detected DDR1 expression in human colon cancer cell lines by FCM. It turned out that seven of nine tested cell lines expressed DDR1 at different levels, except for SW620 and LoVo cell lines with almost no DDR1 expression (Fig. 1C). We also analyzed *DDR1* gene expression by data collected from GDC portal for The Cancer Genome Atlas (GDC‐TCGA)‐CRC. The analysis results, stratified according to the best cut‐off of *DDR1* expression, showed that the high *DDR1* gene expression group had a significantly shorter OS in colon cancer (*P* = 0.046), whereas no correlation between *DDR1* expression and OS was observed in rectum cancer (*P* = 0.3; Fig. S1).

Minimal or no specific staining of DDR1 was recorded in most human normal tissues, e.g. heart, liver, spleen, lung, kidney, cerebrum, cerebellum, bone marrow, thymus, lymph (Fig. 1D). A low to moderate level of positive staining was identified predominantly in stomach, esophagus and mucus of small intestine. Expression of DDR1 in normal tissues was restricted to fast‐growing epithelial cells, particularly in the gastrointestinal tract.

Taking together, these results confirmed the potential of DDR1 as a target for the treatment of colon cancer.

### 3.2 Generation and characterization of the anti‐DDR1 monoclonal antibody

**Figure 2:**
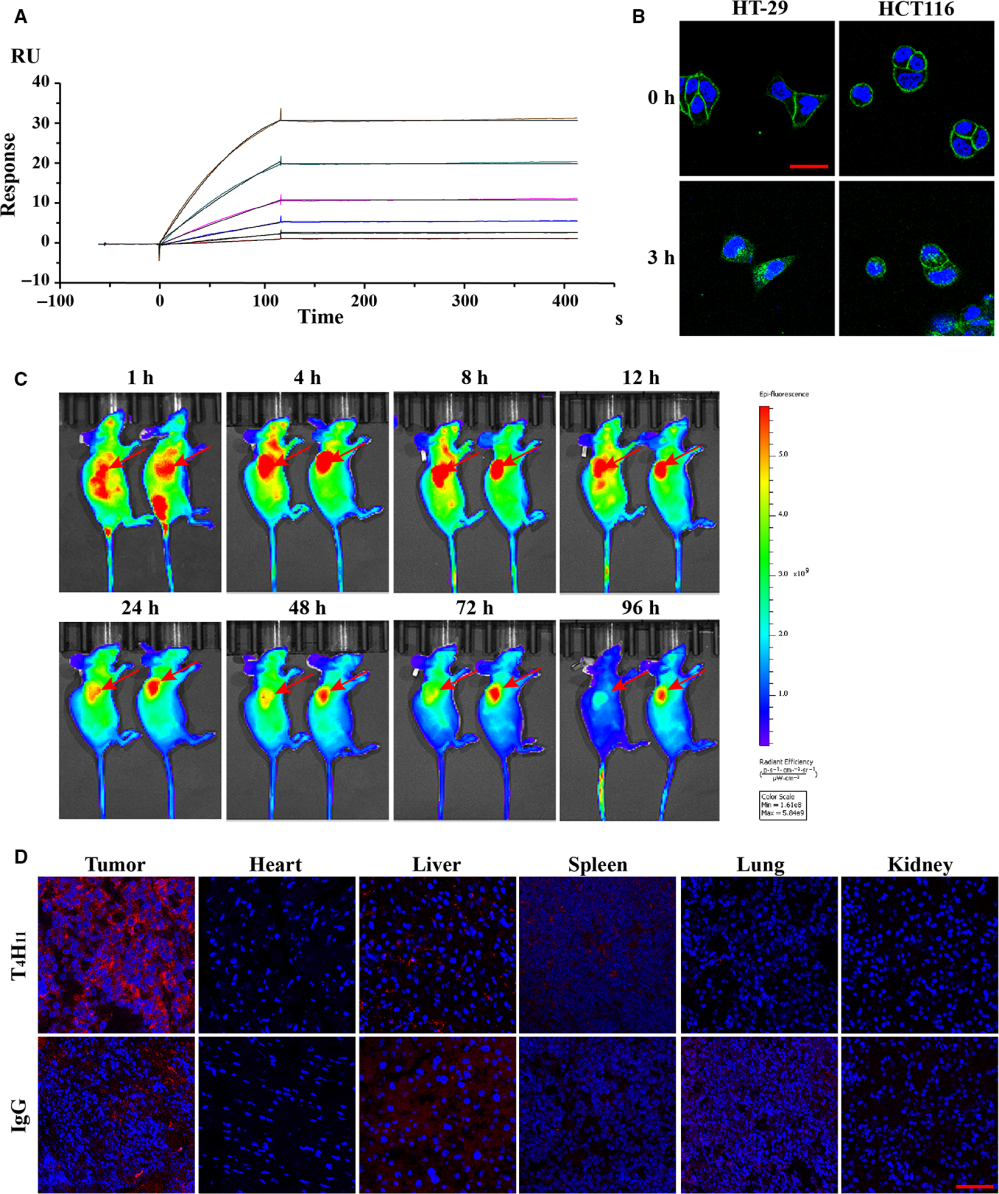
Characterization of T_4_H_11_. (A) Affinity analysis of the antibody T_4_H_11_ using Biacore (*K*_D_ = 2.536 × 10^−10^ mol·L^−1^). (B) Confocal imaging of DDR1 localization and internalization in HT‐29 and HCT116 cells. Cells were treated at 4 °C or 37 °C with 10 μg·mL^−1^ of fluorescein‐coupled T_4_H_11_ and detected by confocal fluorescence microscopy. Green indicates DDR1, and blue indicates DAPI (4′,6‐diamidino‐2‐phenylindole)‐stained nuclear DNA. Red scale bar: 10 μm. (C) *In vivo* fluorescence imaging of BALB/c nude mice bearing subcutaneous HT29 xenografts after intravenous injection with control Cy5.5‐IgG (left) or Cy5.5‐T_4_H_11_ (right) at 10 mg·kg^−1^ at different time points post injection. Red arrows point to tumor. (D) Biodistribution of Cy5.5‐IgG and Cy5.5‐T_4_H_11_ in tumors and vital organs (heart, liver, spleen, lung and kidney) of mice at 12 h post injection. Red scale bar: 70 μm.

Using recombinant His‐DDR1 ECD protein as immunogen and mouse hybridoma technology, a panel of anti‐DDR1 mAbs was generated. The internalization and affinity ability of some candidate antibodies are shown in Figs S2 and S3 and Table S1, respectively. Among these candidates, we selected one mAb, clone T_4_H_11_, based on its high affinity, efficient internalization and absence of cross‐reactivity with DDR1‐related family member DDR2. Besides, T_4_H_11_ bound to the discoidin (DS) domain of DDR1, while did not interfere with interaction of collagen and DDR1 (data not shown) (Abdulhussein *et al*., 2004; Carafoli *et al*., 2012; Leitinger, 2003). T_4_H_11_ antibody was used to stain a panel of frozen tissues from humans by IHC. The results revealed that most normal tissues exhibited negligible staining, except for stomach, esophagus and mucus of small intestine tissues (Fig. S4). The binding kinetics of T_4_H_11_ was measured using SPR (Biacore T200). Fitting of binding curves revealed a strong apparent functional affinity (*K*_D_ = 2.536 × 10^−9^ mol·L^−1^) with fast association (*k*_a _= 1.437 × 10^5^ Ms^−1^) and slow dissociation (*k*_d_ = 3.646 × 10^−5^ s^−1^) rates of T_4_H_11_ (Fig. 2A, Table S2). T_4_H_11_ bound to His‐DDR1 ECD protein but not His‐DDR2 ECD protein (Fig. S5) by ELISA. The *in vitro* cell binding affinity was measured at different concentrations of T_4_H_11_ to cell‐surface DDR1 of HT‐29 cells by FCM (Fig. S6). In addition, cellular trafficking of T_4_H_11_ showed that it bound to the membrane of HT‐29 and HCT116 cells at 4 °C and could be internalized into cells after 3 h incubated at 37 °C, demonstrating reduced membrane staining and increased cytoplasm staining by immunofluorescence (Fig. 2B). In conclusion, T_4_H_11_ had high affinity, specific antigen selectivity and efficient internalization degree *in vitro*.

Next, to clarify the tumor‐binding activity and biodistribution characteristics of T_4_H_11_, the *in vivo* fluorescent images of tumor‐bearing mice were observed by a non‐invasive NIR optical imaging technique. T_4_H_11_‐Cy5.5 and IgG‐Cy5.5 were intravenously injected into tumor‐bearing mice. As shown in Fig. 2C, the fluorescence signals in the T_4_H_11_‐Cy5.5 group were observed in tumor within 1 h post‐injection and were clearly differentiated from the surrounding tissues after 4 h. Fluorescence signals gradually accumulated and remained up to 96 h in the subcutaneous tumor site. Conversely, the fluorescence signals in IgG‐Cy5.5 group were distributed sporadically and were not observed to be as strong as T_4_H_11_‐Cy5.5 in tumor tissues.

To confirm biodistribution of T_4_H_11_‐Cy5.5 in mice, tumors and vital organs (heart, liver, spleen, lung and kidney) were collected at 12 h post injection. Fluorescence signals in these tissues were detected using confocal microscope. There was a strong staining in tumors, a slight staining in liver tissues and no staining in other normal organs of T_4_H_11_‐Cy5.5 (Fig. 2D). Moreover, in tumor tissues, antibodies mainly accumulated in the cytoplasm. These results demonstrated that T_4_H_11_ effectively targeted and located to tumor tissues *in vivo*.

All results confirmed that T_4_H_11_ antibody was a good vehicle for drug delivery in ADC development.

### 3.3 Preparation and characterization of T_4_H_11_‐DM4

**Figure 3:**
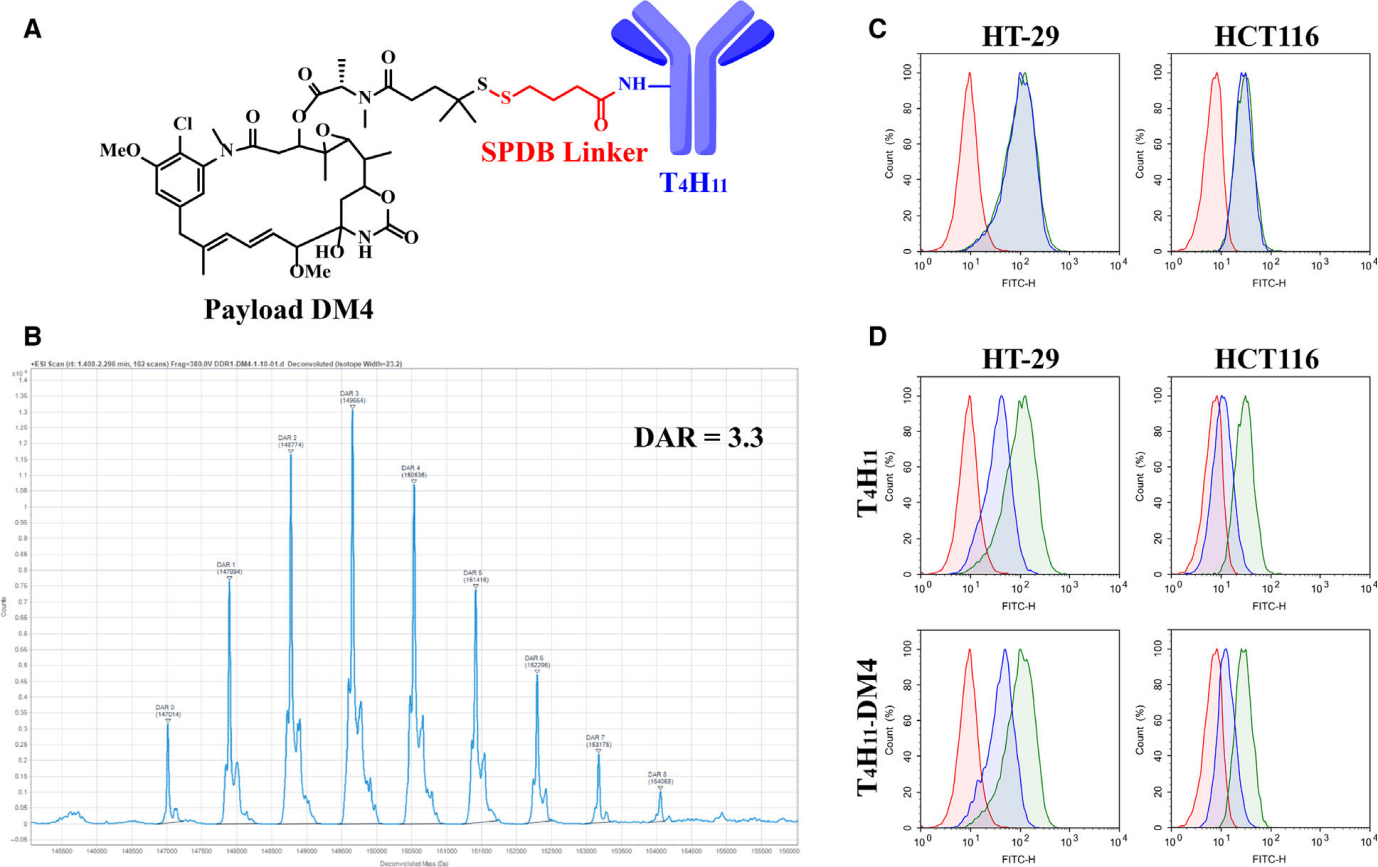
Characterization of T_4_H_11_‐DM4. (A) Structure of the T_4_H_11_‐DM4 consisting of anti‐DDR1 antibody (T_4_H_11_), cleavable disulfide linker SPDB (red) and payload DM4 (black). (B) DAR of T_4_H_11_‐DM4 was determined by LC‐MS (DAR = 3.3). (C,D) FCM analysis was performed to assess the binding and internalization ability of T_4_H_11_ and T_4_H_11_‐DM4 in HT‐29 and HCT116 cell lines. Cells were incubated with unconjugated T_4_H_11_ or T_4_H_11_‐DM4, respectively. The red represents cells incubated with isotype IgG; the green with T_4_H_11_ or T_4_H_11_‐DM4 remained on ice; the blue with T_4_H_11_ or T_4_H_11_‐DM4 shifted to 37 °C for 3 h.

The highly potent microtubule inhibitor DM4 was conjugated to T_4_H_11_ by linker SPDB to produce anti‐DDR1 ADC, named as T_4_H_11_ *‐*DM4 (Fig. 3A). Conventional lysine conjugation method was applied to conjugate SPDB‐DM4 to lysine residues exposed at the surface of T_4_H_11_. The average DAR value was 3.3 mol·mol^−1^ using LC‐MS methods (Fig. 3B). Binding and internalization abilities of T_4_H_11_‐DM4 were confirmed in HT‐29 and HCT116 cell lines. T_4_H_11_‐DM4 bind to tumor cells as effectively as T_4_H_11_, as determined by FCM analysis (Fig. 3C). Also, results from FCM showed that internalization efficiency following cell‐surface binding of T_4_H_11_‐DM4 was similar to that of T_4_H_11_ (Fig. 3D). The internalization rate of T_4_H_11_ and T_4_H_11_‐DM4 was respectively 65 and 61% in HT‐29 cell line and 64 and 67% in HCT116 cell line.

### 3.4 DDR1 ADC exhibits potent and specific cytotoxic activity *in vitro*

**Figure 4:**
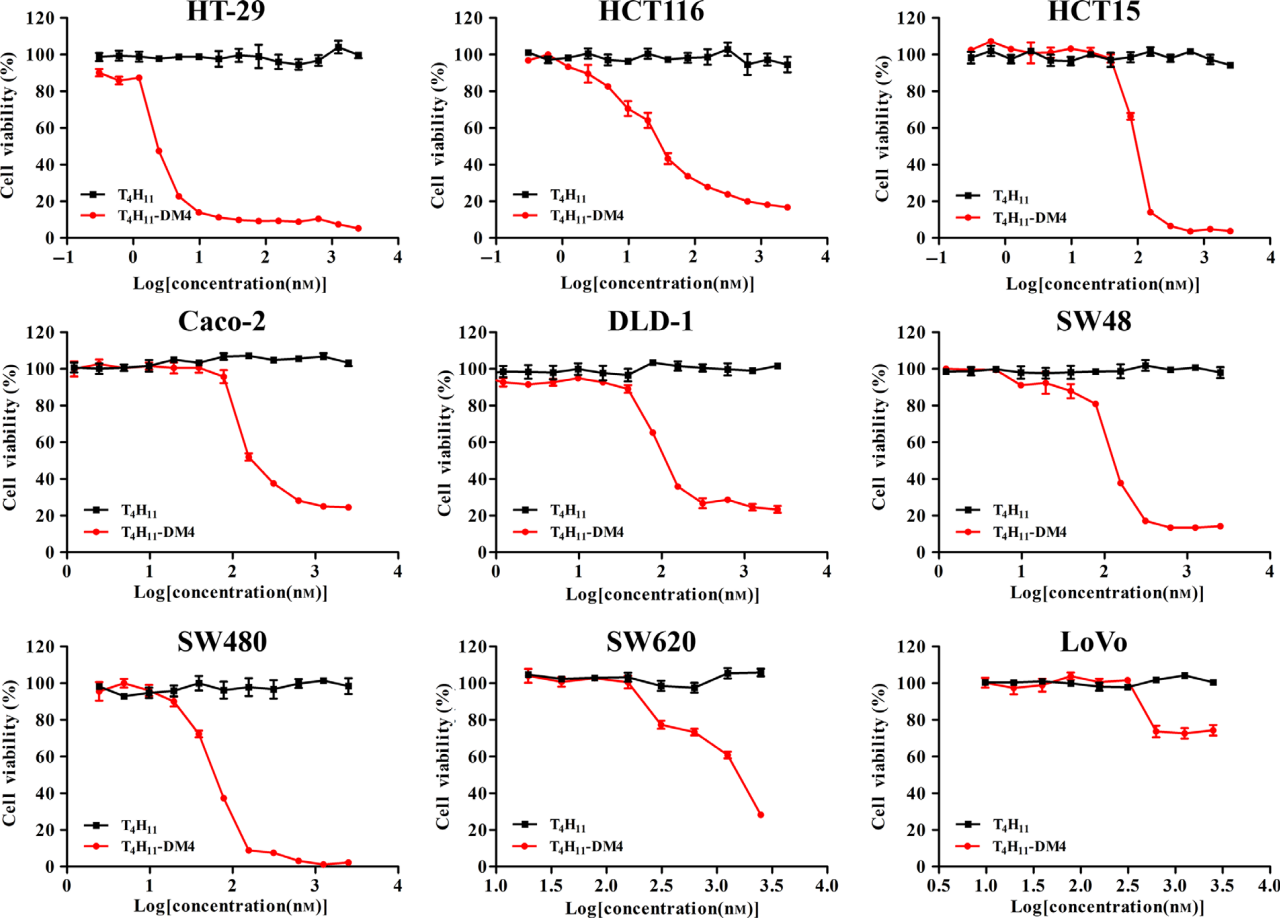
*In vitro* cytotoxicity assays of T_4_H_11_ and T_4_H_11_‐DM4. Cell viability was measured after 72 h after treatment with T_4_H_11_ (solid square; black) or T_4_H_11_‐DM4 (solid circle; red) at several concentrations using CCK‐8. T_4_H_11_‐DM4 but not T_4_H_11_ induced a strong cytotoxicity in DDR1 cell surface expression colon cancer cell lines including HT‐29, HCT116, HCT15, Caco‐2, DLD‐1, SW48 and SW480 cells, respectively. In the DDR1‐negative SW620 and LoVo cell lines, neither treatment had any inhibitory effect. Error bars represent the standard error of the mean.

To evaluate the cell‐killing potency of this novel conjugate, a panel of colon cancer cell lines were incubated with increasing concentrations of T_4_H_11_ or T_4_H_11_‐DM4 for 72 h, after which CCK‐8 was added to analyze cell survival (Fig. 4). The results indicated that T_4_H_11_‐DM4 possesses a strong cytotoxicity *in vitro* among colon cancer cells; IC_50_ values are shown in Table S3. HT‐29 cell line was the most sensitive to T_4_H_11_‐DM4 with the lowest IC_50_ of 2.5 nm. The IC_50_ values of T_4_H_11_‐DM4 in HCT116 and HCT15 cells were 22.1 and 89.4 nm, respectively. Other colon cancer cell lines with varying surface expression levels of DDR1 exhibited IC_50_ ranging from 60.6 to 135.3 nm. Moreover, T_4_H_11_‐DM4 had little effect on DDR1 low or no expression cell lines SW620 and LoVo with IC_50_ > 1 μm. The data demonstrated an overall correlation between DDR1 surface expression and T_4_H_11_‐DM4 cell‐killing activity (Table S1). Unconjugated T_4_H_11_ as well as IgG‐DM4 did not induce cytotoxicity (IC_50_ > 1 μm), indicating that *in vitro* cytotoxicity of T_4_H_11_‐DM4 results from the delivery of payloads rather than from the efficacy of the antibody (Fig. S7).

### 3.5 DDR1 ADC induces significant tumor regression in colon cancer xenografts

**Figure 5:**
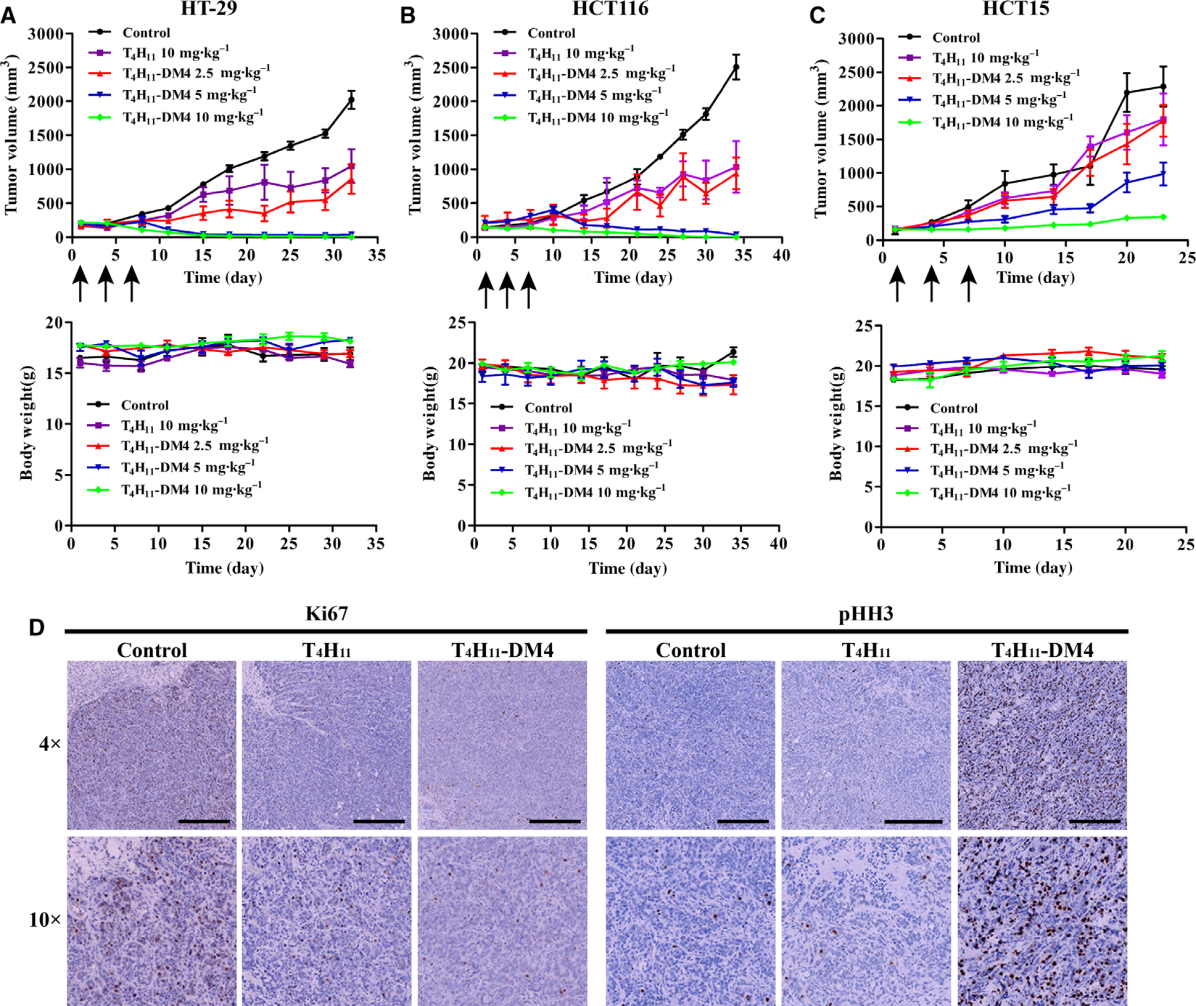
*In vivo* evaluation of T_4_H_11_‐DM4 on tumor growth. (A–C) Antitumor efficacy of T_4_H_11_‐DM4 in HT‐29, HCT116 and HCT15 xenograft models. The tumor‐bearing mice were given PBS (control), T_4_H_11_ (10 mg·kg^−1^) or T_4_H_11_‐DM4 (2.5, 5 or 10 mg·kg^−1^) intravenously on days 1, 4 and 7. Each treatment group included six mice. Each point on the graph represents the average tumor volume. Changes in bodyweight are also represented. Error bars represent standard error of the mean. (D) Mechanisms of the action of T_4_H_11_‐DM4 in HT‐29 xenograft. After three times injections of T_4_H_11_‐DM4 treatment, on day 21 the tumors were harvested and stained with Ki67 antibody (1 : 100 dilution, CST #9027), a marker for proliferative reaction. The number of Ki67‐positive cells significant decreases in T_4_H_11_‐DM4‐treated tumors, indicating an inhibition of tumor proliferation (left). For cell mitosis evaluation, animals bearing HT‐29 tumor xenografts were given a single dose of PBS, T_4_H_11_ (10 mg·kg^−1^) or T_4_H_11_‐DM4 (10 mg·kg^−1^). After 24 h, the tumors were harvested and stained with anti‐phospho‐histone H3 (Ser10) antibody (1 : 100 dilution, CST #9701) to detect mitotic cells (right). The pHH3‐positive tumor cells increased in T_4_H_11_‐DM4 treatment compared with control and T_4_H_11_ group, evidence that DM4 induced cell arrest in mitosis. Black scale bar: 500 μm.

We selected colon cancer cell lines HT‐29, HCT116 and HCT15 expressing different levels of DDR1 to establish mouse subcutaneous tumor models. Mice were injected with different doses of T_4_H_11_‐DM4 or T_4_H_11_ for a total of three injections. The conjugate was found to be highly active in all tested DDR1‐positive xenograft models. Tumors had completely regressed in both HT‐29 and HCT116 tumor models at doses of 5 and 10 mg·kg^−1^ of T_4_H_11_‐DM4 (Fig. 5A,B top). With both doses, the tumor was eliminated for the entire period of therapy. An approximately 60% inhibition in tumor volume was documented at the minimal dose of 2.5 mg·kg^−1^ in HT‐29 and HCT116 xenograft models. There was no significant inhibition in tumor volume between the group using 10 mg·kg^−1^ of control IgG‐DM4 group in HT‐29 xenografts (Fig. S8). In HCT15 tumor model, an obvious delay of tumor growth was observed in a dose‐dependent manner. The average inhibition in tumor volume was 90% for 10 mg·kg^−1^, 60% for 5 mg·kg^−1^ and 22% for 2.5 mg·kg^−1^, respectively (Fig. 5C top). IHC evaluations of *in situ* DDR1 expression in HT‐29 xenografts showed both membrane and cytoplasmic staining (Fig. S9).

All mice behaved normally during the entire observational period. The average bodyweight of treatment groups was comparable to that of control mice with no significant differences (Fig. 5A–C, bottom).

To visualize further the molecular mechanism of anti‐proliferation activity of T_4_H_11_‐DM4, we conducted a pharmacological study in HT‐29 xenografts. Tumors were harvested on day 21 from the 5 mg·kg^−1^ T_4_H_11_‐DM4 treatment group. As expected, the proportion of Ki67‐positive tumor cells appeared marginally greater in antibody or vehicle treating groups than in T_4_H_11_‐DM4 groups (Fig. 5D, left). To assess the intervention of cell mitosis by T_4_H_11_‐DM4, an anti‐phospho‐histone H3 antibody (pHH3; CST, Danvers, MA, USA) was used as a cell‐cycle arrest marker. Tumors were harvested 24 h after a single 10 mg·kg^−1^ dose of T_4_H_11_‐DM4 or 10 mg·kg^−1^ T_4_H_11._ Staining of cells by pHH3 antibody showed that mitotic arrest occurred after treatment of T_4_H_11_‐DM4 but not T_4_H_11_ antibody or vehicle by IHC (Fig. 5D, right). These results demonstrate that T_4_H_11_‐DM4 inhibits proliferation of tumor cells *in vivo* by inducing mitotic arrest in colon xenograft models.

### 3.6 T_4_H_11_‐DM4 potency in platinum‐resistant colorectal cancers

**Figure 6:**
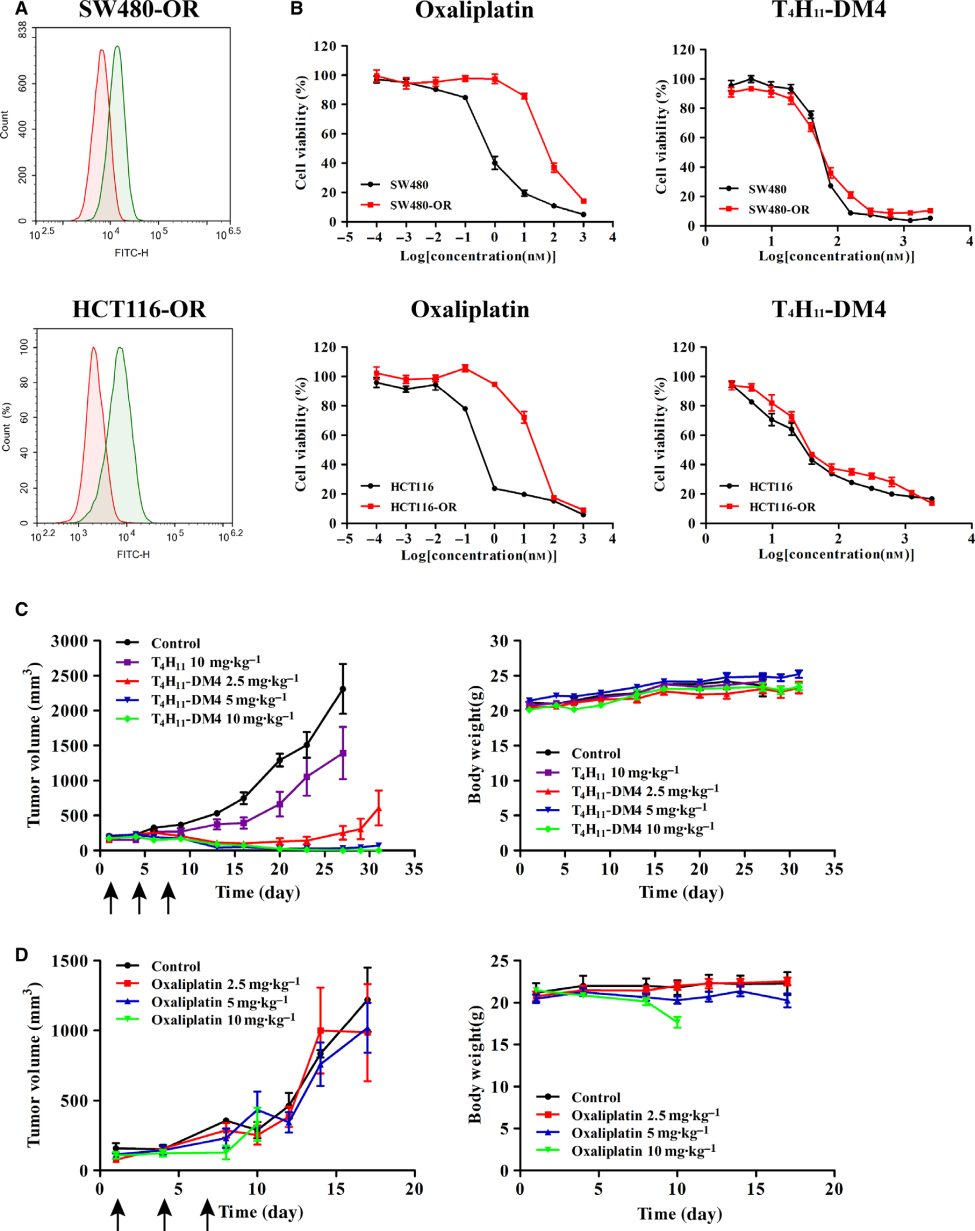
Colorectal cancer cells resistant to platinum were sensitive to T_4_H_11_‐DM4. (A) FCM analysis of DDR1 cell surface expression in SW480‐OR and HCT116‐OR cell lines with an isotype IgG (red) or anti‐DDR1 antibody (green). (B) *In vitro* cytotoxicity of oxaliplatin and T_4_H_11_‐DM4 against SW480‐OR and SW480 (up), HCT116‐OR and HCT116 (down). Error bars represent SD of the mean. (C) Antitumor efficacy of T_4_H_11_‐DM4 in SW480‐OR xenograft model. Animals were dosed once as indicated (arrow) by intravenous injection (Q3D × 3) with T_4_H_11_‐DM4 (2.5, 5 or 10 mg·kg^−1^), T_4_H_11_ (10 mg·kg^−1^) or vehicle (PBS). Error bars represent SD of the mean. (D) Anti‐tumor efficacy of oxaliplatin in SW480‐OR xenograft model. Animals were dosed once as indicated (arrow) by intravenous injection (Q3D × 3) with oxaliplatin (2.5, 5 or 10 mg·kg^−1^) or vehicle (PBS). Error bars represent SD of the mean.

Drug resistance develops in nearly all patients with colon cancer, leading to a decrease in the therapeutic efficacies of anticancer agents. We turned to chemotherapy‐resistant colon cancer cell lines to determine whether DDR1‐ADC demonstrate effective activity. First, DDR1 expression was examined in colon cancer cell lines resistant to oxaliplatin named SW480‐OR and HCT116‐OR by FCM (Fig. 6A). Drug‐resistant cells were 50 times more resistant to oxaliplatin compared with parental cells (Fig. 6B). Notably, T_4_H_11_‐DM4 displayed similar cell proliferation inhibition in both SW480‐OR and HCT116‐OR cell lines with IC_50_ values of 56.9 ± 6.4 nm and 21.2 ± 12.1 nm (Fig. 6B). Furthermore, T_4_H_11_‐DM4 induced complete tumor regressions in SW480‐OR xenograft at 5 and 10 mg·kg^−1^ after three injections, whereas unconjugated mAb or oxaliplatin exhibited little anti‐tumor activity (Fig. 6C,D). These data suggested that the activity of this ADC depended on targeted delivery of cytotoxic drug to DDR1‐expressing cells.

### 3.7 Safety evaluation of T_4_H_11_‐DM4

**Figure 7:**
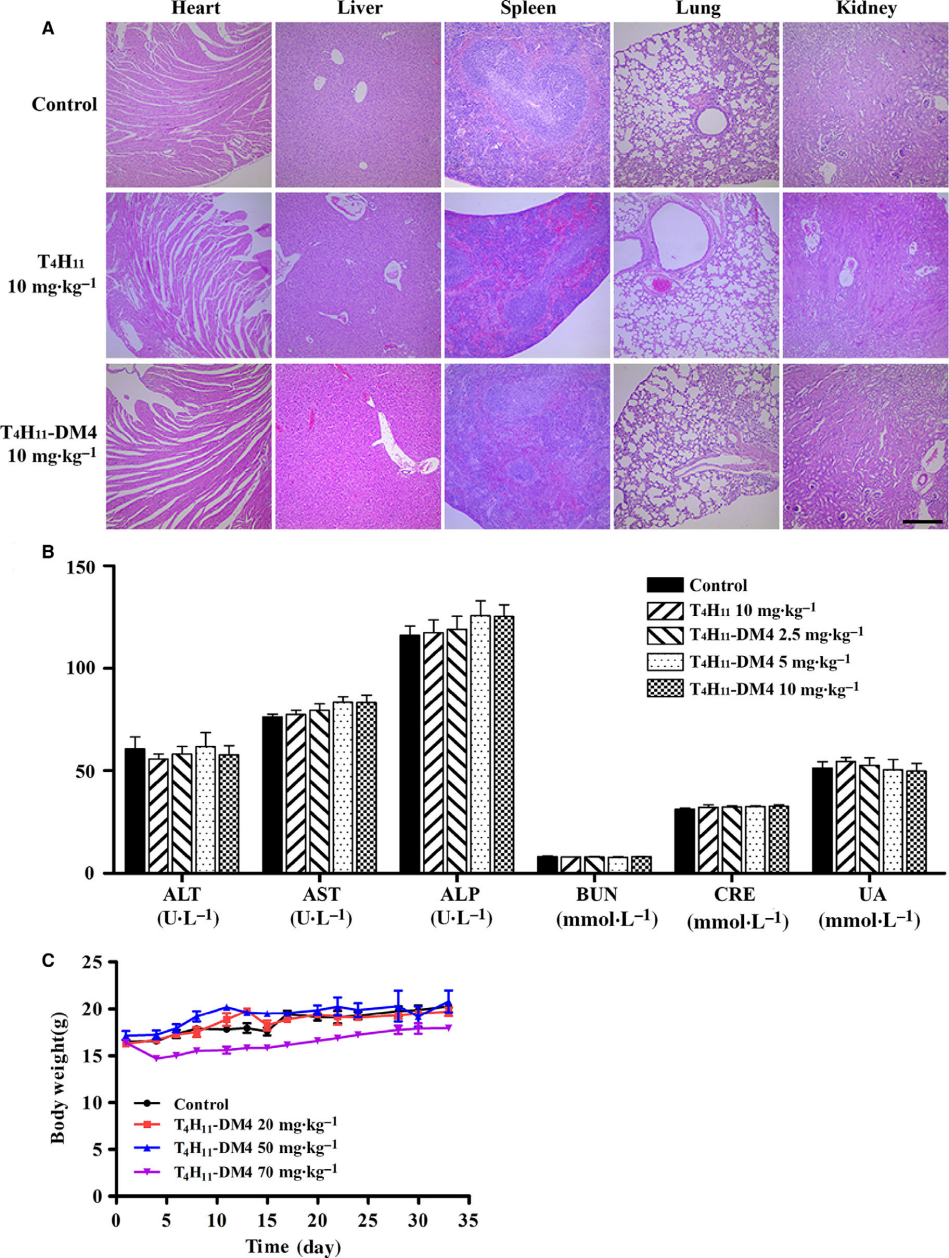
Preliminary safety evaluation of mice receiving T_4_H_11_ or T_4_H_11_‐DM4. (A) H&E images of vital organ of mice at 7 days after treatment with 10 mg·kg^−1^ T_4_H_11,_ T_4_H_11_‐DM4 or PBS for a total of three doses. Magnification: ×100. Black scale bar: 250 μm. (B) The graph depicted ACT, AST, ALP, BUN, CRE, UA 7 days after the last injection. Error bars represent SD of the mean. (C) Bodyweight changes of BALB/c mice after a single‐dose injection of PBS (control) or T_4_H_11_‐DM4 at 20, 50, 70 mg·kg^−1^.

The safety of T_4_H_11_‐DM4 was evaluated using two different types of mice, BALB/c nude mice and BALB/c mice. The first experiment conducted a safety evaluation of multi‐doses of T_4_H_11_‐DM4. BALB/c nude mice received three doses with the vehicle, T_4_H_11_ 10 mg·kg^−1^ or T_4_H_11_‐DM4, at 2.5, 5 or 10 mg·kg^−1^ by intravenous injection. Mice were euthanized 7 days after the last injection; gross pathologic and histopathologic evaluation was performed. Compared with the vehicle group, no significant pathological damages were observed in any doses of T_4_H_11_‐DM4 group by H&E staining of the heart, liver, spleen, lung and kidney as well as biochemical analysis (Fig. 7A,B). In xenograft models of nude mice, therapeutic doses of T_4_H_11_‐DM4 did not induce changes in behavior or bodyweight (Fig. 5A–C, bottom). The second experiment explored a single‐dose injection of T_4_H_11_‐DM4 at 20, 50 or 70 mg·kg^−1^ in BALB/c mice monitored for 33 days. No death occurred among the groups of mice. An average increase was observed in the final bodyweight of mice of about 20% (day 33) compared with the start of the experiment (day 1), and there were no significant differences between experiment groups and control groups, except for the 70 mg·kg^−1^ group, which had an average gain 10% in the final bodyweight of mice (Fig. 7C). A moderate reduction in bodyweight of about 10% was observed in the first 4 days after 70 mg·kg^−1^ of T_4_H_11_‐DM4 injection, but bodyweight recovered during the observation period.

## 4 Discussion

Antibody‐drug conjugates targeting tumor‐specific surface antigens have been clinically proven to be effective treatments for hematologic and solid malignancies (Beck *et al*., 2017). In this study, we show DDR1, an RTK, is a promising candidate target for ADC therapy for colon carcinoma. Our data demonstrated that a novel anti‐DDR1 ADC potently and selectively killed DDR1‐positive colon cancer cells *in vitro* and eliminated the DDR1‐positive colon carcinoma in xenograft models.

Receptor tyrosine kinases are a family of cell surface receptors which regulates many key processes including cell growth and survival (Lemmon and Schlessinger, 2010). Several members of this receptor family (HER2, EGFR, VEGFR, etc.) have become important targets for clinical anti‐cancer therapies (Regad, 2015; Seshacharyulu *et al*., 2012; Tai *et al*., 2010), including small molecular inhibitors and antibody‐based therapies. HER2‐targeting ADC (Ado‐trastuzumab–emtansine, Kadcyla®) was approved for the treatment of breast cancer patients in 2013. Therefore, cancer‐related RTK become preferable targets for ADC development, e.g. RON, PTK7, FLT3, FGFR2, FGFR3, ERBB3, KIT and EPHA2 (Fauvel and Yasri, 2014; Katoh, 2017).

DDR1 is a unique member of RTK family and was first identified during a search for tyrosine kinase proteins expressed in human breast carcinoma (Jing *et al*., 2018; Johnson *et al*., 1993). It has been reported that DDR1 is significantly overexpressed in several human tumors. Recently, a number of small molecule inhibitors of DDR1 were discovered and investigated for their biological effects in animal models (Li *et al*., 2015). However, selective DDR1 inhibitors displayed limited anti‐tumor effect (Kim *et al*., 2013).

The presence of mutations within DDR1 kinase domain in multiple cancers, such as non‐small cell lung cancer (Ford *et al*., 2007) and acute myeloid leukemia (Tomasson *et al*., 2008), which may result in the resistance to small molecular inhibitors, has been reported. In addition, of five DDR1 isoforms, two are kinase‐deficient receptors for missing lacking kinase domain or kinase inactive (Kothiwale *et al*., 2015), which may contribute to the inefficient tumor suppression ability of small molecular inhibitors targeting kinase domain of DDR1. Unlike the small‐molecule approach, ADC presents an opportunity to target the extracellular region of DDR1, thus avoiding the influence of kinase domain.

We identified that DDR1 has suitable characteristics for development as an ADC target for a novel colon cancer treatment approach, including elevated expression, cell‐surface localization and swift endocytosis. Furthermore, IHC studies showed frequent high expression DDR1 among colon cancer patients with a concomitant restricted normal tissue expression profile (Fig. 1A–C). In addition, earlier studies reported that DDR1 could internalize into endosomes of cells after binding to its ligand (Mihai *et al*., 2009). Therefore, we sought to target DDR1with therapeutic antibodies to assess its potential as a novel ADC therapeutic target in colon cancer.

We generated a panel of DDR1 mAbs. The anti‐DDR1 mAb T_4_H_11_ was selected for drug conjugation based on its unique characteristics. T_4_H_11_ was specific to the extracellular region of DDR1, with minimal cross‐reaction with DDR2. The epitope of T_4_H_11_ is within the DS domain of DDR1 but does not overlap with collagen‐binding sites (data not shown). Results showed that T_4_H_11_ could not induce DDR1 phosphorylation and did not interfere with collagen‐induced DDR1 phosphorylation (data not shown). Upon binding to DDR1 in cell surface, a significant and efficient cellular internalization of T_4_H_11_ occurred. Uptake of T_4_H_11_ into cells within a 3‐h incubation period was also observed by immunofluorescence. Moreover, *in vivo* studies confirmed that T_4_H_11_ preferentially targeted and accumulated in tumor tissue and was retained for up to 96 h. Our data demonstrated effective endocytosis and illustrates T_4_H_11_ would serve as an effective vehicle to deliver cytotoxic drugs selectively to the tumor tissue *in vivo*.

We next conjugated T_4_H_11_ with DM4, linked via the cleavable disulfide SPDB. The payload DM4 is a semisynthetic analog of the antimitotic agent maytansine, inhibits microtubule polymerization by binding to tubulin and then inducing mitotic arrest in cells (Oroudjev *et al*., 2010). DM4 binds and disrupts the microtubule network in quickly proliferating cells. Thereby cancer cells are more sensitive to DM4 than normal cells, minimizing side effects. Ado‐trastuzumab emtansine (T‐DM1) using DM1 as a drug warhead has been approved by the FDA for HER2‐positive breast cancer (Amiri‐Kordestani *et al*., 2014). Following the success of T‐DM1, many novel ADC which employ maytansinoids DM1/DM4 as warheads are currently undergoing preclinical or clinical trials (Chen *et al*., 2017). We demonstrated here that after cytotoxic molecules DM4 attached to T_4_H_11_, the binding and internalization abilities of T_4_H_11_ were not been affected compared with unconjugated antibodies, consistent with the requirement for ADC to internalize to mediate payload delivery.

T_4_H_11_‐DM4 showed potent and selective inhibition of cell proliferation in a DDR1‐expressing dependent manner *in vitro*. We observed that targeted delivery of DM4 progressively decreases cell viability 72 h after T_4_H_11_‐DM4 treatment. The reduction in cell viability is dose‐dependent, with IC_50_ values of 2.5–135.3 nm among the DDR1 surface expression‐positive colon cancer cell lines. Results from mouse xenograft colon cancer models proved that T_4_H_11_‐DM4 highly efficiently inhibited tumor growth. *In vivo* studies confirmed that T_4_H_11_‐DM4 inhibited the growth of DDR1‐expressing human colon cancer tumor xenografts models with three doses of T_4_H_11_‐DM4 ADC of 2.5–10 mg·kg^−1^. Inhibition of cell proliferation also occurred by inducing mitotic arrest. Moreover, a correlation between efficacy of T_4_H_11_‐DM4 *in vivo* and levels of DDR1 expression on the cell surface was observed.

Oxaliplatin is the only platinum analog drug that is applied in both first‐line and adjuvant CRC treatment. Acquired resistance to oxaliplatin affects the outcomes of metastatic CRC patients and is commonly observed clinically (Martinez‐Balibrea *et al*., 2015). In our study, DDR1‐positive human colon cancer xenografts resistant to oxaliplatin were eliminated by T_4_H_11_‐DM4 *in vivo*, which indicated that the combination therapy of T_4_H_11_‐DM4 and other small molecular inhibitor might be good option to overcome chemotherapy‐resistance.

Safety and a therapeutic window are very important for ADC. T_4_H_11_‐DM4 exhibited acceptable safety profiles in preliminary animal studies. Within the ECD, mouse DDR1 shares the same amino acid sequence with human DDR1. Therefore, we conducted safety studies in both BALB/c nude mice and BALB/c mice (Fig. 7). Our results showed that T_4_H_11_‐DM4 was relatively safe at therapeutic doses in BALB/c nude mice and was well tolerated in BALB/c mice up to 50 mg·kg^−1^, with a minimal effect on animal behavior and bodyweight. However, a single dose of T_4_H_11_‐DM4 at 70 mg·kg^−1^ caused a reduction of about 10% bodyweight of BALB/c mice. This dose limitation could be a valuable reference for preclinical safety testing in non‐rodent species. DS domain in human DDR1 shares the same amino acid sequences with cyno DDR1*,* suggesting that T_4_H_11_ could bind to cyno DDR1, offering a convenient way to evaluate safety of T_4_H_11_‐DM4 in monkey. Moreover, preclinical toxicity, pharmacology and pharmacokinetics‐pharmacodynamics evaluation in monkey will be conducted after humanization and validation of antibody conjugate.

Taking into consideration results from tissue cross‐reactivity studies of T_4_H_11_ by IHC, expression of DDR1 in normal gastrointestinal tract tissues indicate that the major side effects of T_4_H_11_‐DM4 may be gastrointestinal system toxicity. The ideal targeting antigens for ADC‐based cancer therapies are tumor‐specific, but these kinds of antigens are relatively rare. Tyrosine kinase receptor Her 2, the target of FDA‐approved ADC T‐DM1, is highly expressed in many cancers such as breast cancer, gastric cancer and ovarian cancer. However, Her 2 is also expressed in some normal tissues such as heart and gastrointestinal tissues, which lead to cardiotoxic side effects and gastrointestinal disorders during T‐DM1 treatment (Kowalczyk *et al*., 2017). There is a significant difference in DDR1 expression between tumor tissues and normal tissues. Therefore, anti‐DDR1 ADC probably has a certain safety and therapeutic window for targeted cancer therapies.

In summary, anti‐DDR1 ADC was highly efficacious in DDR1‐expressing colon cancer xenograft models and exhibited acceptable safety profiles in preliminary animal studies, suggesting that anti‐DDR1 ADC may be a promising therapeutic for the treatment of patients with colon cancer. However, some issues about the DDR1‐targeted ADC should be further studied. T_4_H_11_ was specific to the DS domain of DDR1, but the exact binding epitope of T_4_H_11_ remains unknown (Leitinger, 2003). DDR1 is a collagen receptor that mediates cell–microenvironment communication in tumors. Whether anti‐DDR1 ADC can coordinate with checkpoint immunotherapy for tumor deserves further research (Gadiya and Chakraborty, 2018).

## 5 Conclusion

In summary, T_4_H_11_‐DM4 showed significant tumor growth inhibition and its toxicity was tolerable within the therapeutic dose range in mice models. Our data showed that DDR1‐based ADC could be a promising therapeutic agent for colon cancer. Although these results are encouraging, more investigation needs to be done to assess whether DDR1‐based ADC is effective and safe for clinical application.

## Conflict of interest

The authors declare no conflict of interest.

## Author contributions

YT and RW contributed equally to this work. YT, RW and JY conceived and designed the experiments. YT, RW, YWa, XJ and YL developed and verified the analytical methods. YT, MW, TY, YF, WL, YP and LZ acquired the data. YT, RW, SZ, ZL and LG analyzed and interpreted the data. YT, RW and QL wrote the manuscript. YY, YWu and LY provided technical supports. ZZ, CG and GZ assisted in preparing laboratory appliance and reagents. All authors contributed to revision of the manuscript and approved the final version for publication.

## Supporting information


**Fig. S1.** Survival curves of colon cancer and rectum cancer patients with high and low *DDR1* expression status in TCGA dataset. *DDR1* gene expression and survival data were acquired by GDC portal for the cancer genome atlas (GDC‐TCGA). The cutoff of *DDR1* expression was identified by the best cutoff (Youden Index) in ROC analysis for death detection.

**Fig. S2.** Internalization rate of some candidate antibodies. Names of antibodies are as follows: R5‐E12‐C3, T2‐C8‐G12, T3‐D11‐H5, R1‐A6‐H8, T1‐C10‐C2, Y4‐D4‐F7, Y4‐D4‐G11 and T4‐C2‐C5. The red represents cells incubated with control IgG; the green with each antibody remained on ice; the blue with the corresponding antibody shifted to 37 ℃ for 3 h.

**Fig. S3.** Kinetic analysis of candidate anti‐DDR1 monoclonal antibodies to recombinant human DDR1 ECD by SPR. Names of antibodies are as follows: R5‐E12‐C3, T2‐C8‐G12, T3‐D11‐H5, R1‐A6‐H8, T1‐C10‐C2, Y4‐D4‐F7, Y4‐D4‐G11 and T4‐C2‐C5. Each antibody was assayed in a 2‐fold serial dilution with concentrations of 2 nm, 4 nm, 8 nm, 16 nm, 32 nm and 64 nm.

**Fig. S4.** Representative images of T_4_H_11_ staining for DDR1 expression in human normal tissues. 1, cerebrum; 2, cerebellum; 3, heart; 4, liver; 5, spleen; 6, lung; 7, kidney; 8, spinal cord; 9, nerve; 10, lymph node; 11, adrenal gland; 12, skeletal muscle; 13, smooth muscle; 14, ovary; 15, testis, 16, stomach; 17, esophagus; 18, small intestine; 19, colon; 20, nerve; 21, salivary gland; 22, thyroid gland. Magnification, × 10. Black scale bar: 250 μm.

**Fig. S5.** Binding ability of antibody to recombinant proteins. T_4_H_11_ was detected for DDR1 family member cross‐reactivity by ELISA. DDR1 ECD (dot, black) or DDR2 ECD (square, red) was coated onto an ELISA plate. T_4_H_11_ was applied at the indicated concentrations. Error bars represent the standard error of the mean (SEM).

**Fig. S6**. Antibody *in vitro* binding ability for living cells. Cells expressing DDR1 at the surface (HT‐29) were incubated with T_4_H_11_ over a range of concentrations prior to staining with Alexa Fluor 488‐labeled goat anti‐mouse IgG (H+L) secondary antibody. Mean fluorescence intensity (MFI) of Alexa Fluor 488 signal was measured by FCM.

**Fig. S7.** Inhibition of *in vitro* cell proliferation by T_4_H_11_‐DM4 and control IgG‐DM4. Cell viability was measured at 72 h after treatment with T_4_H_11_‐DM4 (solid square; red) or IgG‐DM4 (solid circle; black) at several concentrations in HT‐29 colon cancer cells using CCK‐8 assay. Cell viability was profoundly inhibited by T_4_H_11_‐DM4. The IC_50_ value of T_4_H_11_‐DM4 and IgG‐DM4 were 4.57 ± 2.07 nm and more than 1000 nm, respectively. Error bars represent the standard error of the mean (SEM).

**Fig. S8.** *In vivo* antitumor efficacy of T_4_H_11_‐DM4 and IgG‐DM4 against HT‐29 xenografts. Antitumor efficacy of T_4_H_11_‐DM4 and IgG‐DM4 in HT‐29 xenograft models (*n* = 6/group). The tumor‐bearing mice were given PBS (control), IgG‐DM4 or T_4_H_11_‐DM4 intravenously on days 1, 4 and 7 for three total doses after tumors were established. Each point on the graph represents the average tumor volume. Both T_4_H_11_‐DM4 (solid triangle; green) and IgG‐DM4 (solid squares; red) were dosed at 10 mg/kg. Changes in bodyweight are also represented. Error bars represent the standard error of the mean (SEM).

**Fig. S9.** Representative images of IHC staining for DDR1 in HT‐29 xenograft tumor tissues. Cells of HT‐29 were injected s.c. into nude mice. Tumor from control group was removed and processed for IHC and stained for DDR1 expression as described in the supporting methods. Scale bar: (left) 200 μm, (right) 40 μm.

**Table S1.** Kinetic association (K_a_) and dissociation parameters (K_d_), along with calculated affinity (K_D_) values of some candidate antibodies measured by Biacore.

**Table S2.** Kinetic association (K_a_) and dissociation parameters (K_d_), along with calculated affinity (K_D_) were measured of T_4_H_11_ or T_4_H_11_‐DM4 by Biacore.

**Table S3.** *In vitro* potency of T_4_H_11_‐DM4 in colon cancer cell lines with different cell surface expression levels of DDR1.
