# Supplementary figures and images for: Targeting of DDR1 with antibody‐drug conjugates has antitumor effects in a mouse model of colon carcinoma
Source: Mol Oncol. 2019 Jul 22;13(9):1855–73. doi: 10.1002/1878-0261.12520 (PMC6717758; doi:10.1002/1878-0261.12520)

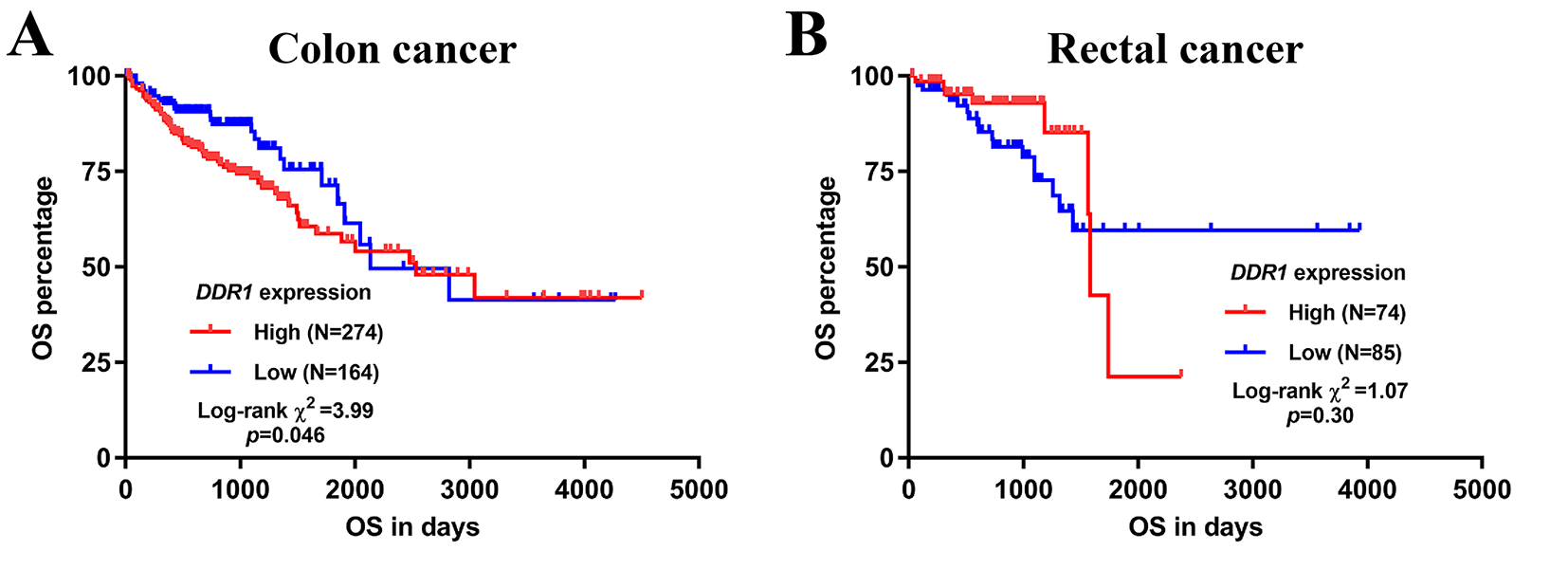

Supplement: Supplementary file 1 — Fig. S1. Survival curves of colon cancer and rectum cancer patients with high and low DDR1 expression status in TCGA dataset. DDR1 gene expression and survival data were acquired by GDC portal for the cancer genome atlas (GDC‐TCGA). The cutoff of DDR1 expression was identified by the best cutoff (Youden Index) in ROC analysis for death detection. [file MOL2-13-1855-s001.tif]

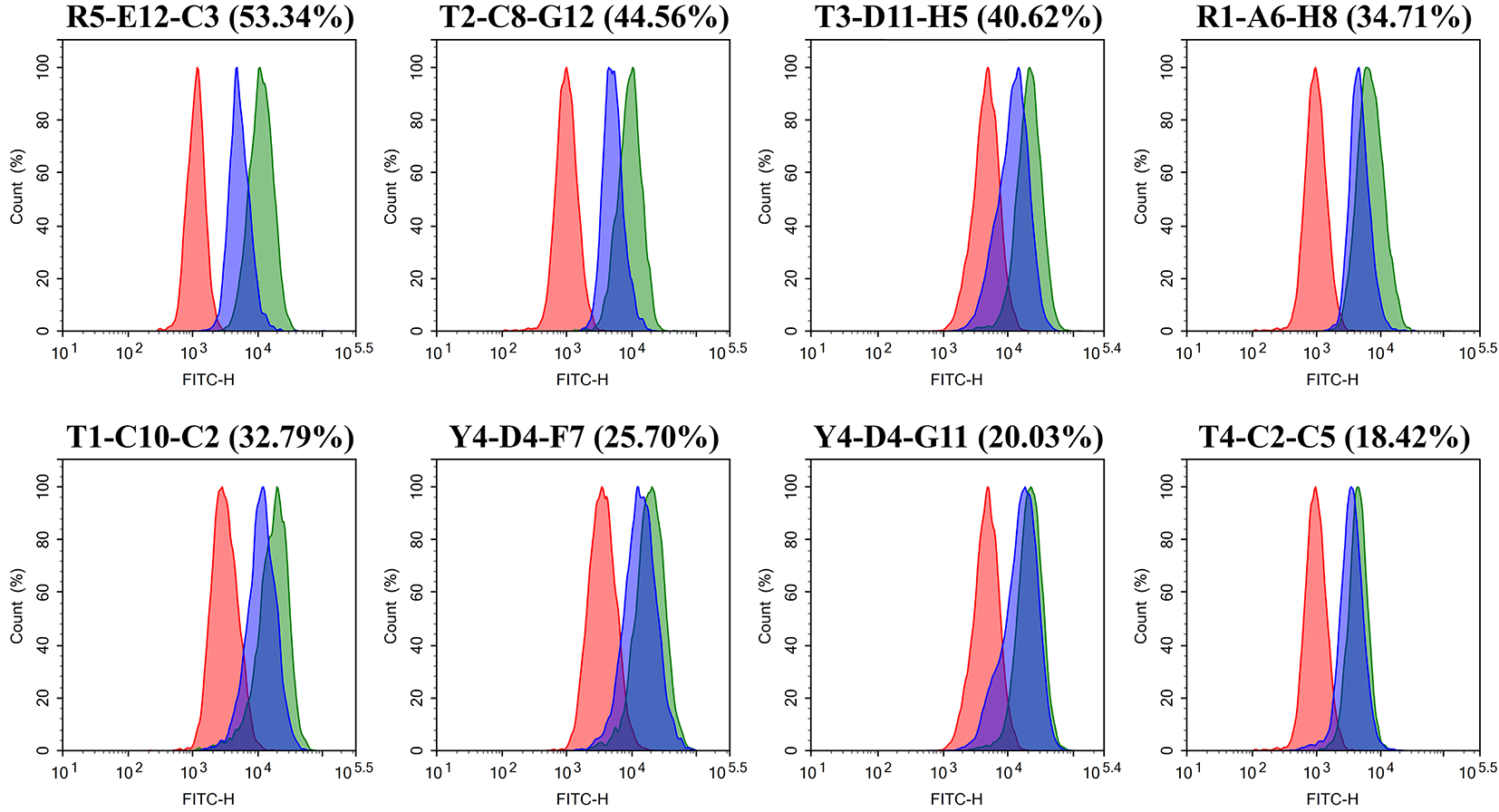

Supplement: Supplementary file 2 — Fig. S2. Internalization rate of some candidate antibodies. Names of antibodies are as follows: R5‐E12‐C3, T2‐C8‐G12, T3‐D11‐H5, R1‐A6‐H8, T1‐C10‐C2, Y4‐D4‐F7, Y4‐D4‐G11 and T4‐C2‐C5. The red represents cells incubated with control IgG; the green with each antibody remained on ice; the blue with the corresponding antibody shifted to 37 ℃ for 3 h. [file MOL2-13-1855-s002.tif]

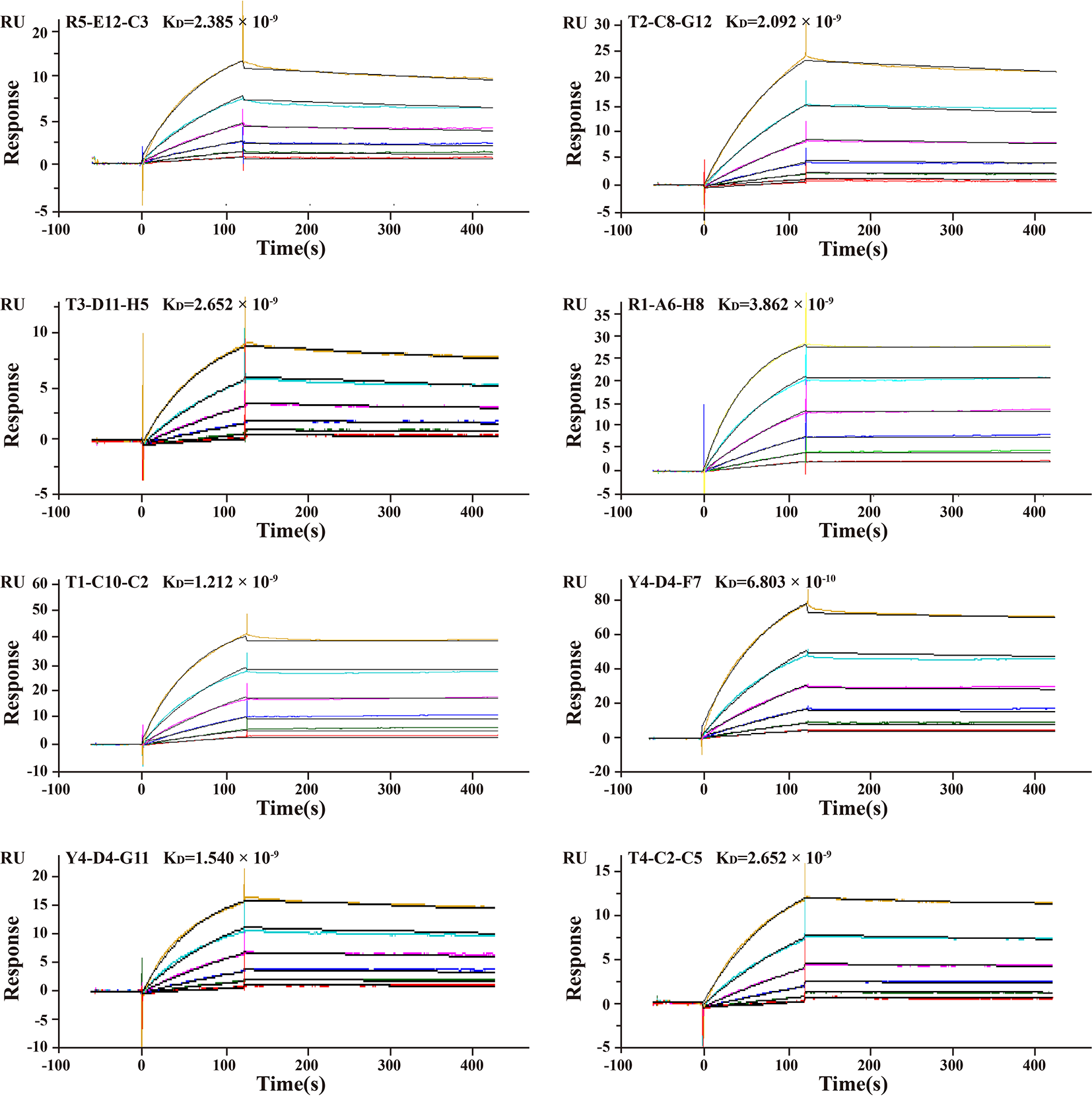

Supplement: Supplementary file 3 — Fig. S3. Kinetic analysis of candidate anti‐DDR1 monoclonal antibodies to recombinant human DDR1 ECD by SPR. Names of antibodies are as follows: R5‐E12‐C3, T2‐C8‐G12, T3‐D11‐H5, R1‐A6‐H8, T1‐C10‐C2, Y4‐D4‐F7, Y4‐D4‐G11 and T4‐C2‐C5. Each antibody was assayed in a 2‐fold serial dilution with concentrations of 2 nm, 4 nm, 8 nm, 16 nm, 32 nm and 64 nm. [file MOL2-13-1855-s003.tif]

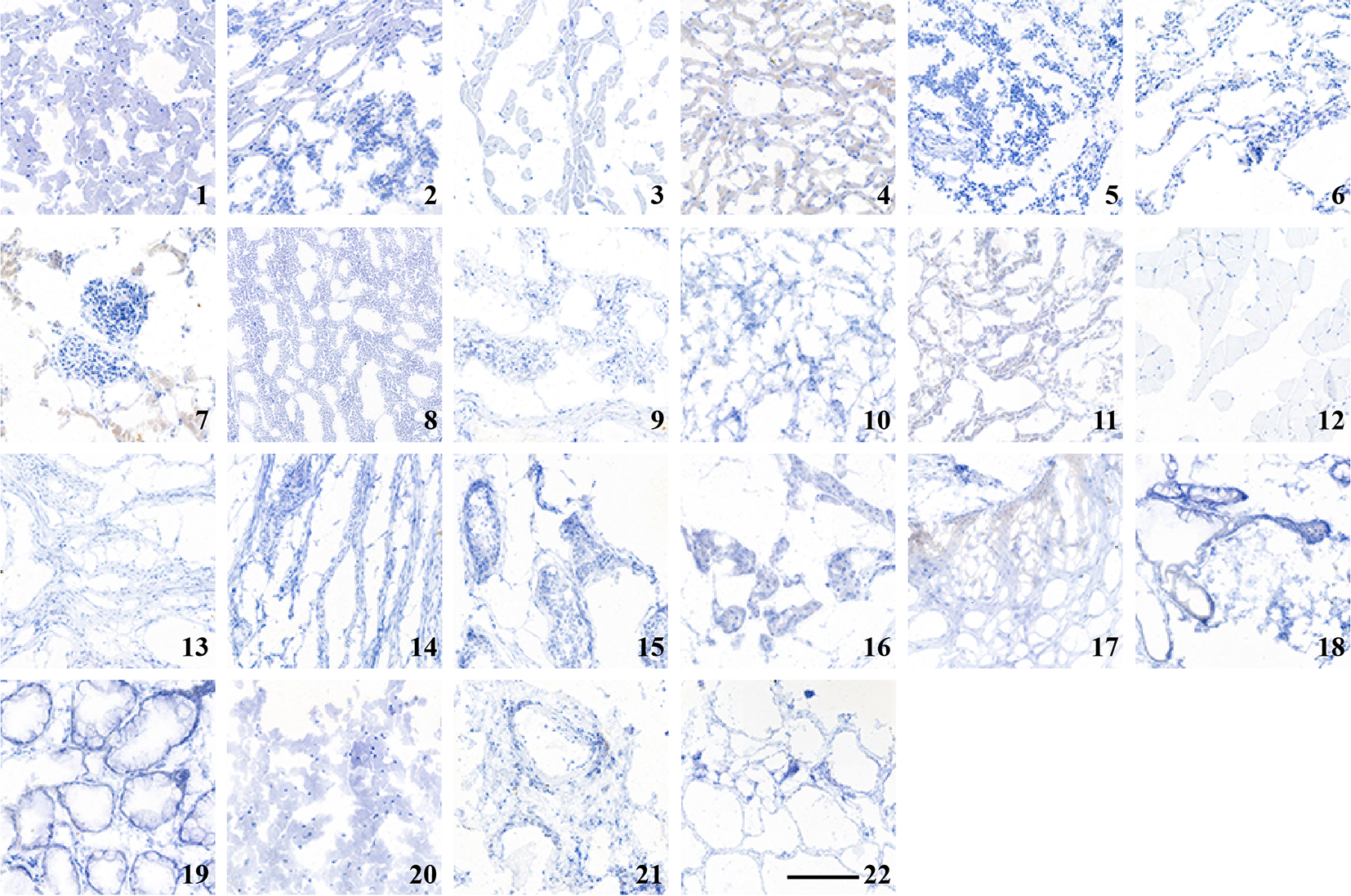

Supplement: Supplementary file 4 — Fig. S4. Representative images of T4H11 staining for DDR1 expression in human normal tissues. 1, cerebrum; 2, cerebellum; 3, heart; 4, liver; 5, spleen; 6, lung; 7, kidney; 8, spinal cord; 9, nerve; 10, lymph node; 11, adrenal gland; 12, skeletal muscle; 13, smooth muscle; 14, ovary; 15, testis, 16, stomach; 17, esophagus; 18, small intestine; 19, colon; 20, nerve; 21, salivary gland; 22, thyroid gland. Magnification, × 10. Black scale bar: 250 μm. [file MOL2-13-1855-s004.tif]

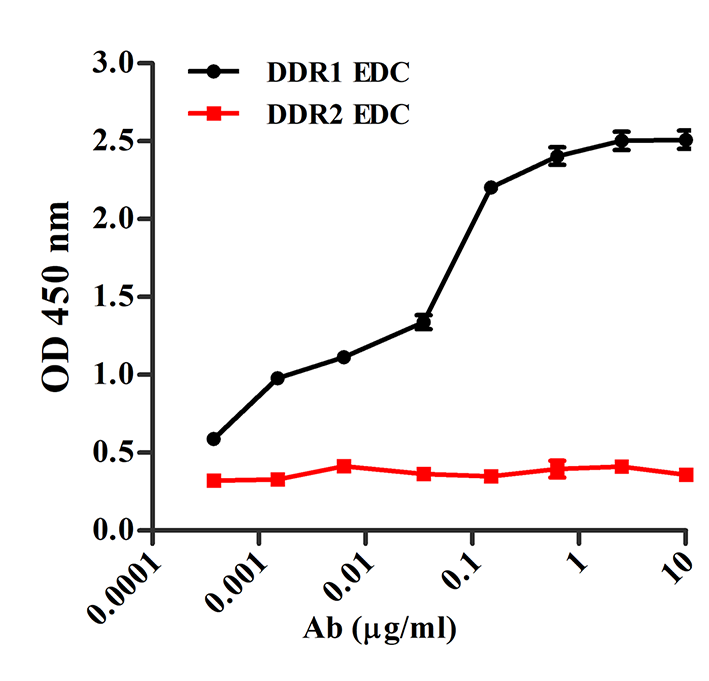

Supplement: Supplementary file 5 — Fig. S5. Binding ability of antibody to recombinant proteins. T4H11 was detected for DDR1 family member cross‐reactivity by ELISA. DDR1 ECD (dot, black) or DDR2 ECD (square, red) was coated onto an ELISA plate. T4H11 was applied at the indicated concentrations. Error bars represent the standard error of the mean (SEM). [file MOL2-13-1855-s005.tif]

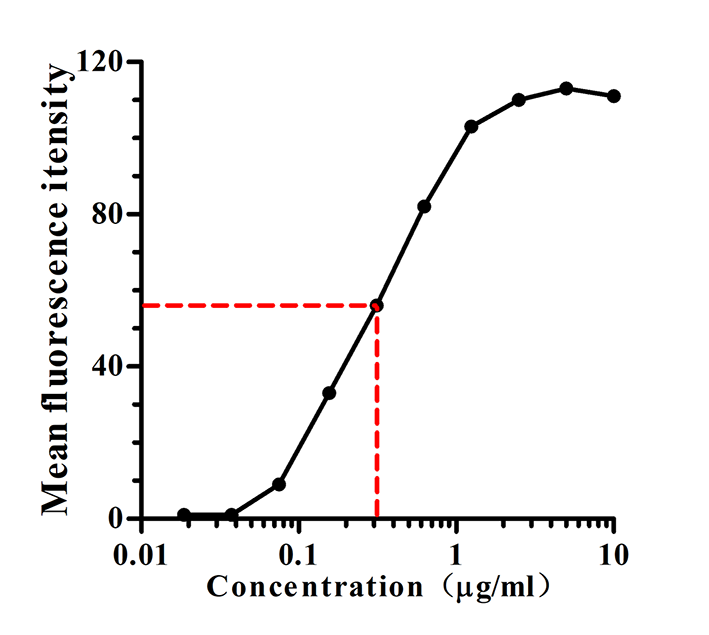

Supplement: Supplementary file 6 — Fig. S6. Antibody in vitro binding ability for living cells. Cells expressing DDR1 at the surface (HT‐29) were incubated with T4H11 over a range of concentrations prior to staining with Alexa Fluor 488‐labeled goat anti‐mouse IgG (H+L) secondary antibody. Mean fluorescence intensity (MFI) of Alexa Fluor 488 signal was measured by FCM. [file MOL2-13-1855-s006.tif]

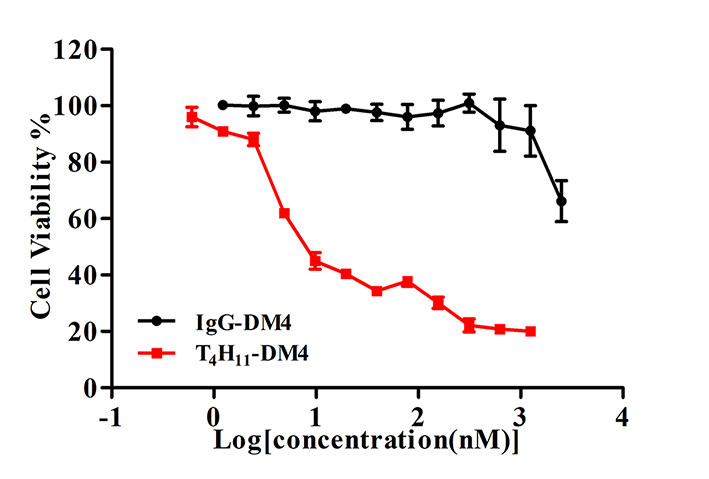

Supplement: Supplementary file 7 — Fig. S7. Inhibition of in vitro cell proliferation by T4H11‐DM4 and control IgG‐DM4. Cell viability was measured at 72 h after treatment with T4H11‐DM4 (solid square; red) or IgG‐DM4 (solid circle; black) at several concentrations in HT‐29 colon cancer cells using CCK‐8 assay. Cell viability was profoundly inhibited by T4H11‐DM4. The IC50 value of T4H11‐DM4 and IgG‐DM4 were 4.57 ± 2.07 nm and more than 1000 nm, respectively. Error bars represent the standard error of the mean (SEM). [file MOL2-13-1855-s007.tif]

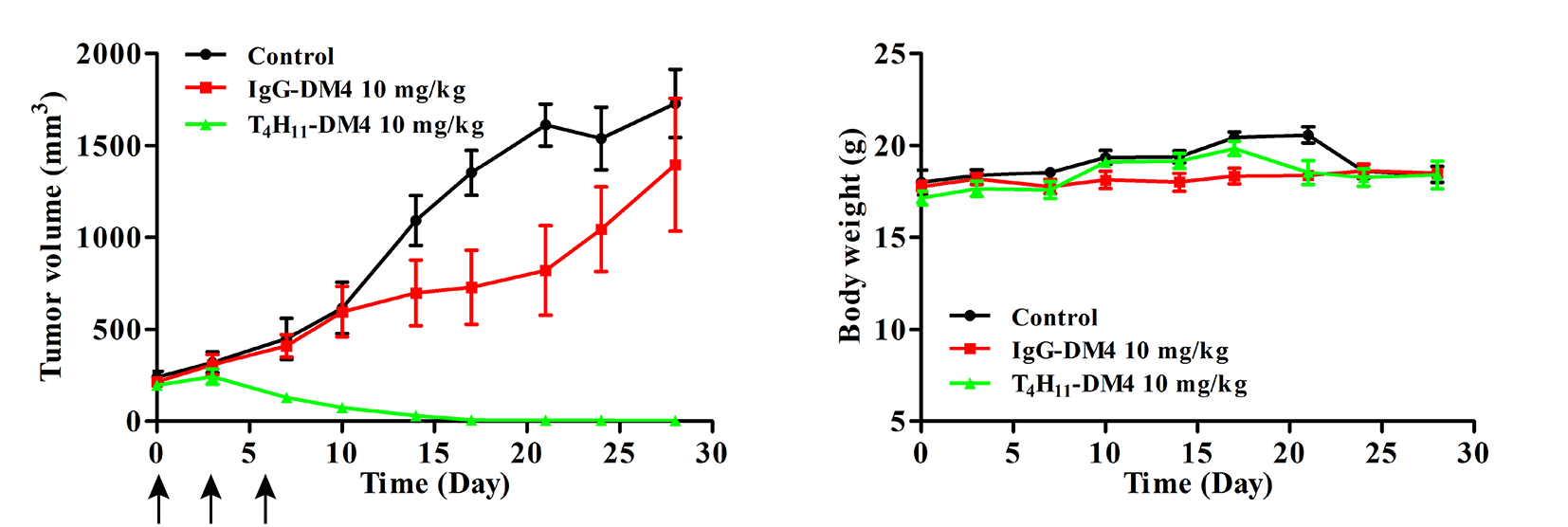

Supplement: Supplementary file 8 — Fig. S8. In vivo antitumor efficacy of T4H11‐DM4 and IgG‐DM4 against HT‐29 xenografts. Antitumor efficacy of T4H11‐DM4 and IgG‐DM4 in HT‐29 xenograft models (n = 6/group). The tumor‐bearing mice were given PBS (control), IgG‐DM4 or T4H11‐DM4 intravenously on days 1, 4 and 7 for three total doses after tumors were established. Each point on the graph represents the average tumor volume. Both T4H11‐DM4 (solid triangle; green) and IgG‐DM4 (solid squares; red) were dosed at 10 mg/kg. Changes in bodyweight are also represented. Error bars represent the standard error of the mean (SEM). [file MOL2-13-1855-s008.tif]

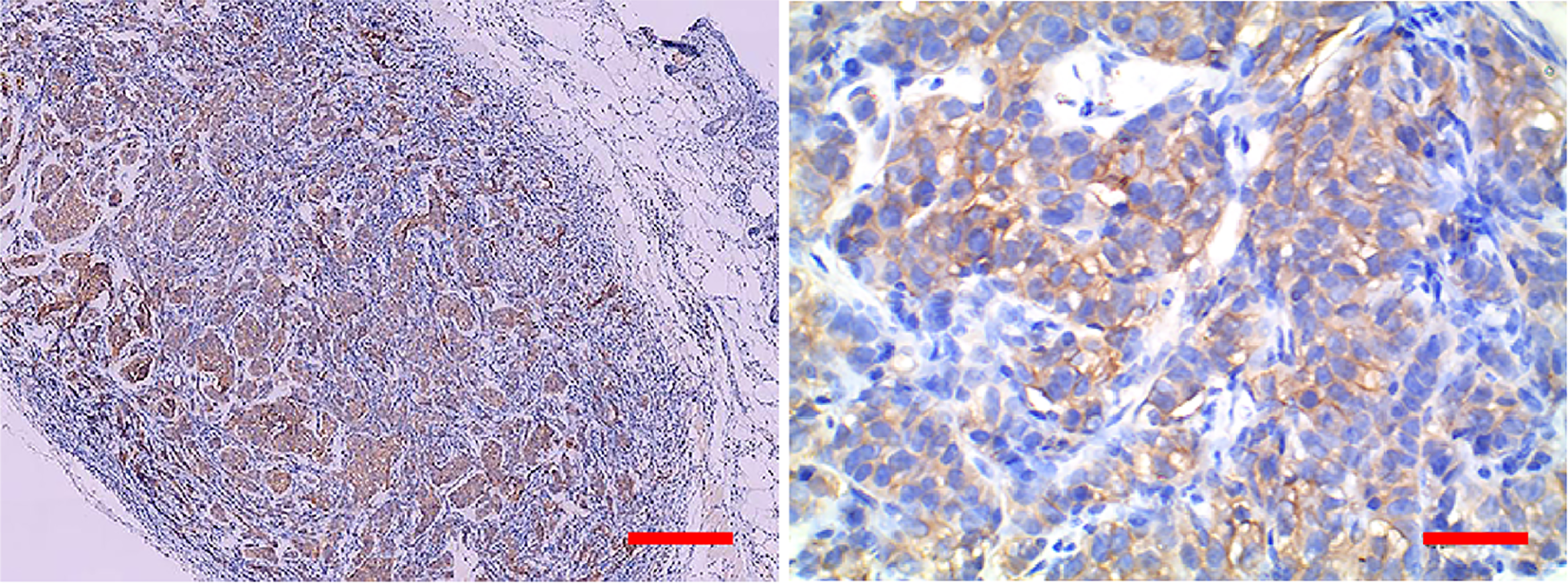

Supplement: Supplementary file 9 — Fig. S9. Representative images of IHC staining for DDR1 in HT‐29 xenograft tumor tissues. Cells of HT‐29 were injected s.c. into nude mice. Tumor from control group was removed and processed for IHC and stained for DDR1 expression as described in the supporting methods. Scale bar: (left) 200 μm, (right) 40 μm. [file MOL2-13-1855-s009.tif]
