# Supplementary material for: Targeting of DDR1 with antibody‐drug conjugates has antitumor effects in a mouse model of colon carcinoma
Source: Mol Oncol. 2019 Jul 22;13(9):1855–73. doi: 10.1002/1878-0261.12520 (PMC6717758; doi:10.1002/1878-0261.12520)
Supplement: Supplementary file 10 — Table S1. Kinetic association (Ka) and dissociation parameters (Kd), along with calculated affinity (KD) values of some candidate antibodies measured by Biacore. [file MOL2-13-1855-s010.docx]

**Supporting Information Table S1.** Kinetic association (K_a_) and dissociation parameters (K_d_), along with calculated affinity (K_D_) values of some candidate antibodies measured by Biacore.

| **Antibody** | **K_a_ (M^-1^s^-1^)** | **K_d_ (s^-1^)** | **K_D_ (nM)** |
| --- | --- | --- | --- |
| R5-E12-C3 | 1.875 × 10^5^ | 4.472 × 10^-4^ | 2.385 |
| T2-C8-G12 | 1.514 × 10^5^ | 3.167 × 10^-4^ | 2.092 |
| T3-D11-H5 | 1.911 × 10^5^ | 5.068 × 10^-4^ | 2.652 |
| R1-A6-H8 | 2.956 × 10^5^ | 11.41× 10^-4^ | 3.862 |
| T1-C10-C2 | 2.428 × 10^5^ | 2.943 × 10^-4^ | 1.212 |
| Y4-D4-F7 | 2.067 × 10^5^ | 4.663 × 10^-5^ | 0.226 |
| Y4-D4-G11 | 2.224 × 10^5^ | 3.425 × 10^-4^ | 1.540 |
| T4-C2-C5 | 1.570 × 10^5^ | 1.936 × 10^-4^ | 1.233 |
