# Supplementary material for: Targeting of DDR1 with antibody‐drug conjugates has antitumor effects in a mouse model of colon carcinoma
Source: Mol Oncol. 2019 Jul 22;13(9):1855–73. doi: 10.1002/1878-0261.12520 (PMC6717758; doi:10.1002/1878-0261.12520)
Supplement: Supplementary file 11 — Table S2. Kinetic association (Ka) and dissociation parameters (Kd), along with calculated affinity (KD) were measured of T4H11 or T4H11‐DM4 by Biacore. [file MOL2-13-1855-s011.docx]

**Supporting Information Table S2.** Kinetic association (K_a_) and dissociation parameters (K_d_), along with calculated affinity (K_D_) were measured of T_4_H_11_ or T_4_H_11_-DM4 by Biacore.

| **Antibody** | **K_a_ (M^-1^s^-1^)** | **K_d_ (s^-1^)** | **K_D_ (nM)** |
| --- | --- | --- | --- |
| T_4_H_11_ | 1.437 × 10^5^ | 3.646 × 10^-5^ | 2.536 |
| T_4_H_11_-DM4 | 1.472 × 10^5^ | 3.173 × 10^-5^ | 2.156 |
