# Supplementary material for: Targeting of DDR1 with antibody‐drug conjugates has antitumor effects in a mouse model of colon carcinoma
Source: Mol Oncol. 2019 Jul 22;13(9):1855–73. doi: 10.1002/1878-0261.12520 (PMC6717758; doi:10.1002/1878-0261.12520)
Supplement: Supplementary file 12 — Table S3. In vitro potency of T4H11‐DM4 in colon cancer cell lines with different cell surface expression levels of DDR1. [file MOL2-13-1855-s012.docx]

**Supporting Information Table S3.** *In vitro* potency of T_4_H_11_-DM4 in colon cancer cell lines with different cell surface expression levels of DDR1

| **Tumor Cells** | **Relative expression**  **of DDR1** | **IC_5_** ± **SEM（nM）** | | |
| --- | --- | --- | --- | --- |
|  |  | **T_4_H_11_-DM4** | **T_4_H_11_** | **Oxaliplatin** |
| HT-29 | 11.2 | 2.5±0.02 | >1000 | nd |
| HCT116 | 2.8 | 22.1±0.9 | >1000 | 0.3 ±0.03 |
| HCT15 | 1.9 | 89.4±2.7 | >1000 | nd |
| SW48 | 3.0 | 120.5±4.7 | >1000 | nd |
| Caco-2 | 8.0 | 135.3±7.5 | >1000 | nd |
| DLD-1 | 3.2 | 88.1±4.2 | >1000 | nd |
| SW480 | 3.3 | 60.6±1.8 | >1000 | 0.7 ± 0.20 |
| SW480-O | 2.2 | 56.9±6.4 | >1000 | 48.4±4.1 |
| HCT116-O | 2.1 | 21.2±12.1 | >1000 | 18.7±2.9 |
| SW620 | 1.2 | >1000 | >1000 | nd |
| LOVO | 1.4 | >1000 | >1000 | nd |

Relative expression of DDR1 presented as MFI values of experimental group samples subtracted that of their respective control group. nd represents no detection.
